# Liver-Specific Expressions of *HBx* and *src* in the *p53* Mutant Trigger Hepatocarcinogenesis in Zebrafish

**DOI:** 10.1371/journal.pone.0076951

**Published:** 2013-10-09

**Authors:** Jeng-Wei Lu, Wan-Yu Yang, Su-Mei Tsai, Yueh-Min Lin, Pen-Heng Chang, Jim-Ray Chen, Horng-Dar Wang, Jen-Leih Wu, Shiow-Lian Catherine Jin, Chiou-Hwa Yuh

**Affiliations:** 1 Institute of Molecular and Genomic Medicine, National Health Research Institutes, Zhunan, Miaoli, Taiwan; 2 Department of Life Sciences, National Central University, Jhongli City, Taoyuan, Taiwan; 3 Department of Pathology, Changhua Christian Hospital, Changhua City, Changhua County, Taiwan; 4 Department of Medical Technology, Jen-Teh Junior College of Medicine, Nursing and Management, Hou-Loung Town, Miaoli County, Taiwan; 5 Department of Veterinary Medicine, National Taiwan University, Taipei, Taiwan; 6 Department of Pathology, Chang Gung Memorial Hospital, Keelung, Taiwan; 7 College of Medicine, Chang Gung University, Taoyuan, Taiwan; 8 Institute of Biotechnology, National Tsing Hua University, Hsinchu, Taiwan; 9 Institute of Cellular and Organismic Biology, Academia Sinica, Nangang District, Taipei, Taiwan; 10 Institute of Bioinformatics and Structural Biology, National Tsing Hua University, Hsinchu, Taiwan; 11 Department of Biological Science and Technology, National Chiao Tung University, Hsinchu, Taiwan; Institute of Cellular and Organismic Biology, Taiwan

## Abstract

Hepatocarcinogenesis is a multistep process that starts from fatty liver and transitions to fibrosis and, finally, into cancer. Many etiological factors, including hepatitis B virus X antigen (HBx) and p53 mutations, have been implicated in hepatocarcinogenesis. However, potential synergistic effects between these two factors and the underlying mechanisms by which they promote hepatocarcinogenesis are still unclear. In this report, we show that the synergistic action of HBx and p53 mutation triggers progressive hepatocellular carcinoma (HCC) formation via src activation in zebrafish. Liver-specific expression of HBx in wild-type zebrafish caused steatosis, fibrosis and glycogen accumulation. However, the induction of tumorigenesis by HBx was only observed in p53 mutant fish and occurred in association with the up-regulation and activation of the src tyrosine kinase pathway. Furthermore, the overexpression of *src* in p53 mutant zebrafish also caused hyperplasia, HCC, and sarcomatoid HCC, which were accompanied by increased levels of the signaling proteins p-erk, p-akt, myc, jnk1 and vegf. Increased expression levels of lipogenic factors and the genes involved in lipid metabolism and glycogen storage were detected during the early stages of hepatocarcinogenesis in the HBx and *src* transgenic zebrafish. The up-regulation of genes involved in cell cycle regulation, tumor progression and other molecular hallmarks of human liver cancer were found at later stages in both HBx and src transgenic, p53 mutant zebrafish. Together, our study demonstrates that HBx and src overexpression induced hepatocarcinogenesis in p53 mutant zebrafish. This phenomenon mimics human HCC formation and provides potential *in vivo* platforms for drug screening for therapies for human liver cancer.

## Introduction

Hepatocellular carcinoma (HCC) ranks as the third leading cause of cancer deaths worldwide [1]. Hepatocarcinogenesis progresses gradually from hepatitis to steatosis, fibrosis, and cirrhosis, before eventually becoming HCC. Many risk factors, such as hepatitis B or C virus infection, exposure to aflatoxin B (AFB1), or p53 mutation, cause liver damage and lead to the development of HCC [1–5]. However, the potential synergistic effects among the risk factors for HCC formation remain elusive.

HBV X (HBx) antigen is the most studied oncogene in HBV; HBx has been shown to enhance colony formation in cell lines [6] and induce HCC in mice [7,8]. In zebrafish, HBx overexpression causes hepatic steatosis and liver degeneration in a wild-type background [9]. The co-expression of HBx and the HCV core protein induces intrahepatic cholangiocarcinoma [10], and combined treatment with HBx and AFB1 triggers liver hyperplasia during the early stages of hepatocarcinogenesis [11]. However, transgenic zebrafish only overexpressing HBx do not develop HCC.

Zebrafish is an excellent animal model for studying liver cancer. Neoplasia can be induced by carcinogens [12–14], and in zebrafish, HCC can be driven by inducible Kras(V16) and mutated EGFR isoforms [15,16]. Zebrafish liver tumors are highly analogous to human tumors in terms of comparative analysis of microarray data and ultrasound biomicroscopy [13,14]. 

Src is a non-receptor tyrosine kinase that displays aberrantly high activity in various human cancers, including HCC [17]. We previously identified the up-regulation of Src as an early-stage HCC biomarker that is important for cancer maintenance in the HBx-induced HCC mouse model [16]. However, whether liver-specific overexpression of src in zebrafish will cause HCC has not been shown. In this study, we report the potential synergism between HBx overexpression and p53 mutation in HCC development and reveal a role for src in HCC progression in transgenic zebrafish. Our study provides insightful pathologic information regarding HCC induced by HBx and src in p53 mutant zebrafish, as well as valuable *in vivo* drug screening platforms for liver cancer therapy.

## Materials and Methods

### Zebrafish maintenance

The zebrafish were maintained at the Zebrafish Core Facility at NTHU-NHRI (ZeTH) according to established protocols [18]. All zebrafish experiments were approved by the Institutional Animal Care and Use Committee (IACUC) of the NHRI (NHRI-IACUC-101005-A) (Experimental Procedures S1).

### Generation of transgenic zebrafish using the Tol2 transposon system

The liver-specific HBx and src transgenic fish were generated as previously described [11,19] (Experimental Procedures S1). 

### Liver tissue collection, paraffin sectioning, and histological and immunohistochemistry analyses

HBx transgenic zebrafish were euthanized at 1.5, 3, 5, 7, 9, and 11 months of age, and src transgenic zebrafish were euthanized at all of the stages listed above except 1.5 months. The liver tissues were frozen in liquid nitrogen immediately after sectioning for RNA preparation or were fixed and embedded in paraffin for histological and immunohistochemistry analysis, as previously described [11] (Experimental Procedures S1). All samples were examined and assessed by three experienced pathologists, Drs. Yueh-Min Lin, Pen-Heng Chang, and Jim-Ray Chen.

### Sirius Red staining, periodic acid-Schiff staining, TUNEL assay, and Oil red O staining

All the staining, including Sirius Red, periodic acid-Schiff, TUNEL assay, and Oil red O staining, were carried out as previously described [11] (Experimental Procedures S1). The scoring method was also described previously [11], and the standard for scoring is provided in Figure S3.

### RNA isolation and quantitative RT-PCR

The isolation of RNA and q-PCR were carried out as previously described [11] (Experimental Procedures S1). The primer sequences are listed in Table S1. 

### Western blot analysis

The western blot analysis method was also described previously [20] (Experimental Procedures S1).

### Statistical analysis

The unpaired Student's t test, Kaplan-Meier analysis, ANOVA test and two-tailed Fisher exact test were used in the data analysis as previously described [11,21] (Experimental Procedures S1). A p-value less than 0.05 was considered to be statistically significant.

## Results

### Liver-specific expression of HBx-mCherry fusion protein in transgenic zebrafish

**Figure 1:**
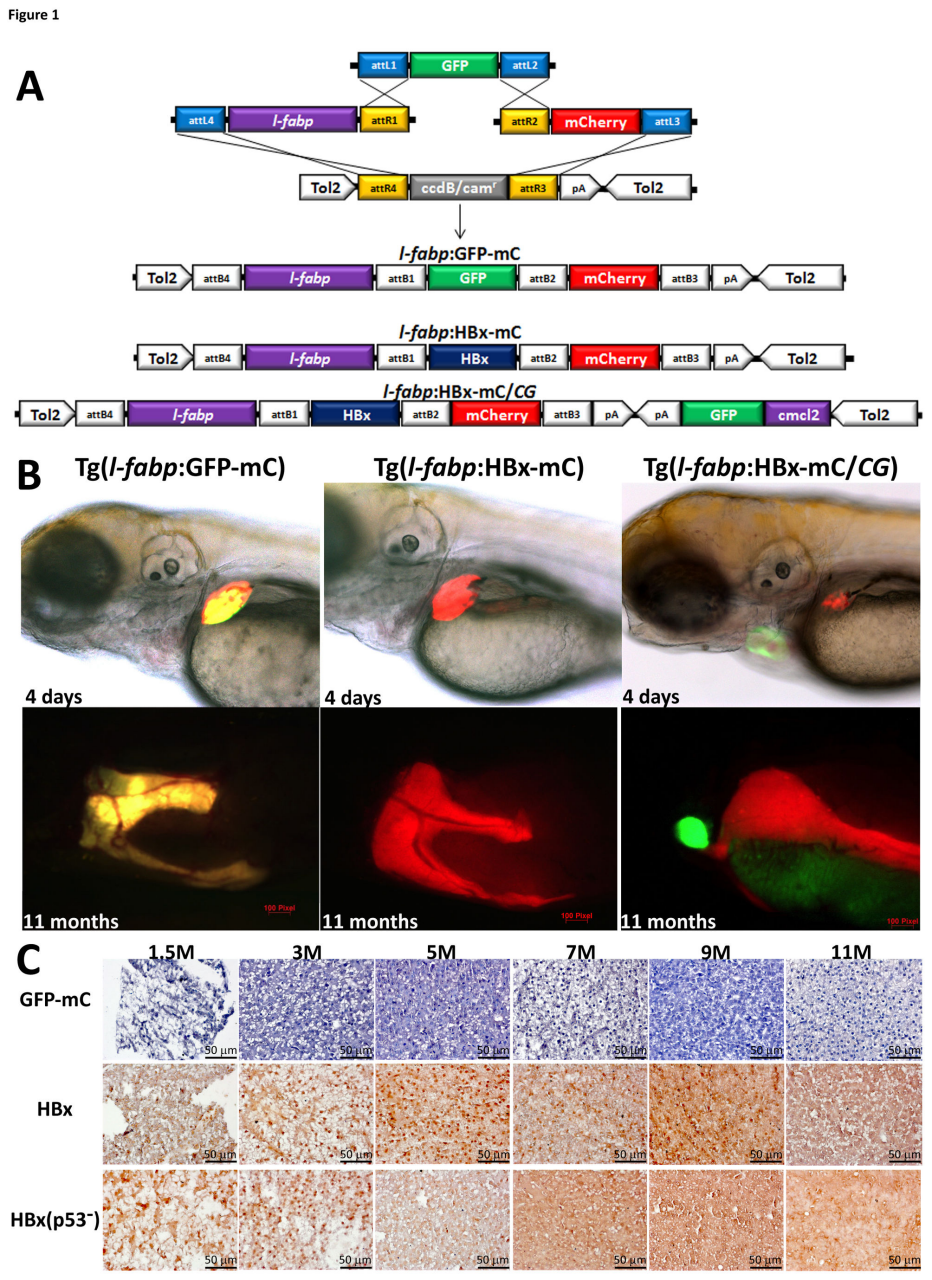
The generation and characterization of the Tg(*l-fabp*:GFP-mCherry), *Tg*(*l-fabp*:HBx-mCherry) and Tg(*l-fabp*:HBx-mCherry;*cmcl2*:GFP) transgenic zebrafish. (A) A schematic diagram of the LR recombination reaction used to generate the expression constructs, including three entry clones (p5E-*l-fabp*, pME-GFP and p3E-mCherry) and two destination vectors (pDestTol2pA and pDestTol2CG2) that contain the *cmlc2*:GFP-pA expression cassette. The three final constructs are shown at the bottom of the figure. (B) The expression of the GFP-mCherry fusion protein and the HBx-mCherry fusion protein in the liver are shown in four-day-old embryos and eleven-month-old adult wild-type fish harboring the *l-fabp*:GFP-mCherry transgene and the *l-fabp*:HBx-mCherry transgene, respectively. The HBx-mCherry fusion protein is expressed in the liver of p53 mutant fish harboring the *l-fabp*:HBx-mCherry;*cmcl2*:GFP transgene, as indicated by the red fluorescence, and *cmlc2*:GFP is expressed in the heart, as indicated by the green fluorescence. (C) HBx protein expression in the hepatocytes was detected via immunostaining of the liver sections of the 1.5-, 3-, 5-, 7-, 9- and 11-month-old fish harboring the *l-fabp*:HBx-mCherry transgene in the wild-type background or the *l-fabp*:HBx-mCherry;*cmcl2*:GFP transgene in the p53 mutant background (x 400). Scale bars: 50 μm.

Three constructs were generated, in which the genes were driven under the control of the liver-specific *fabp10* (fatty acid-binding protein 10) promoter (abbreviated as *l-fabp*) [22] (Figure 1A). The constructs were flanked by the Tol2 transposon element [19] and were co-injected with Tol2 transposase mRNA into one-cell embryos. First, we examined the fluorescence of four-day-old larvae and eleven-month-old adult fish to screen for the expression of the transgene (Figure 1B). The expression of the *l-fabp*:GFP-mC construct was verified by the green and red fluorescence of the GFP-mCherry fusion gene in the liver, which served as a control. Three Tg(*l-fabp*:HBx-mC) founders, which showed red fluorescence from the HBx-mCherry fusion gene in the liver, were generated. Two Tg(*l-fabp*:HBx-mC/*CG*) founders, which showed the red fluorescence of the HBx-mCherry fusion protein in the liver and the green fluorescence of GFP under the control of the heart-specific *cmlc2* promoter in the heart, were generated in the p53 mutant background (Figure 1B). The GFP expression in the heart was used to mark the p53 mutant background. The expression of the HBx protein was verified via immunohistochemistry using an anti-HBx antibody. In contrast to the hepatocytes of the Tg(*l-fabp*:GFP-mC) fish, which showed no HBx expression, the Tg(*l-fabp*:HBx-mC) fish exhibited strong expression levels of the HBx protein from 1.5 to 11 months of age (Figure 1C).

### A synergistic interaction between HBx and the p53 mutation facilitates hyperplasia and HCC formation in the livers of HBx transgenic fish

In the previous reports, HBx transgenic fish in a wild-type background did not develop HCC [9]. Mutations in p53 have been shown to facilitate hepatocarcinogenesis [23]. The zebrafish p53(M214K) mutation is two amino acids away from the most frequently mutated hotspot that is found in human HCC, p53(R249S) [24,25]. To examine whether this p53 mutation facilitates the formation of HCC in HBx-overexpressing transgenic fish, we introduced the liver-specific expression of HBx in wild-type and homozygous p53(M214K) mutant fish (abbreviated as p53^-^). In this experiment, Tg(*l-fabp*:GFP-mC) fish served as controls because they do not have an oncogenic predisposition. Histopathological examination was conducted on hepatocytes from three HBx and two HBx(p53^-^) transgenic lines at six different ages (1.5, 3, 5, 7, 9 and 11 months). A total of 177 liver specimens were analyzed by H&E staining (Table S1). The identification of tissue pathology was performed by three pathologists experienced in carcinogenesis and was based on the criteria set by the National Toxicology Program [26] and those used in recent literature [27]. According to these guidelines, the liver samples were examined and classified into different histopathological categories, representative images of which are shown in Figure S1. In contrast to normal hepatocytes (Figure S1A), upon dissection, fewer liver samples were green, and based on the deposition of yellow-green globular bilirubin pigment after H&E staining, the fish were diagnosed as having cholestasis (Figure S1B). Some HBx or src transgenic fish were found to have clear intracytoplasmic vacuoles during the early stages of the disease; these vacuoles were considerably more extensive than those observed in the control fish. By staining fresh liver sections with Oil red O (Figure S2), we confirmed that these vacuoles were filled with fat, and accordingly, we classified this pathology as steatosis (Figure S1C). Hyperplasia was characterized by the accelerated proliferation of atypical hepatocytes with enlarged and mildly irregular nuclei (Figure S1D), and dysplasia was characterized by transformed cells with enlarged nuclei and prominent nucleoli (Figure S1E). In the later stages, we often found that the hepatocytes were characterized by an enlargement of polymorphic nuclei, prominent nucleoli and an increased number of mitotic figures, and accordingly, these fish were diagnosed as having HCC (Figure S1 F and G). The HCC sometimes co-existed with marked cystic degeneration (spongiosis hepatis) (Figure S1F). We also found that the src transgenic fish developed pleomorphic spindle tumor cells that grew in haphazardly fascicular patterns, and we therefore diagnosed this pathology as sarcomatoid HCC (Figure S1H).

**Figure 2:**
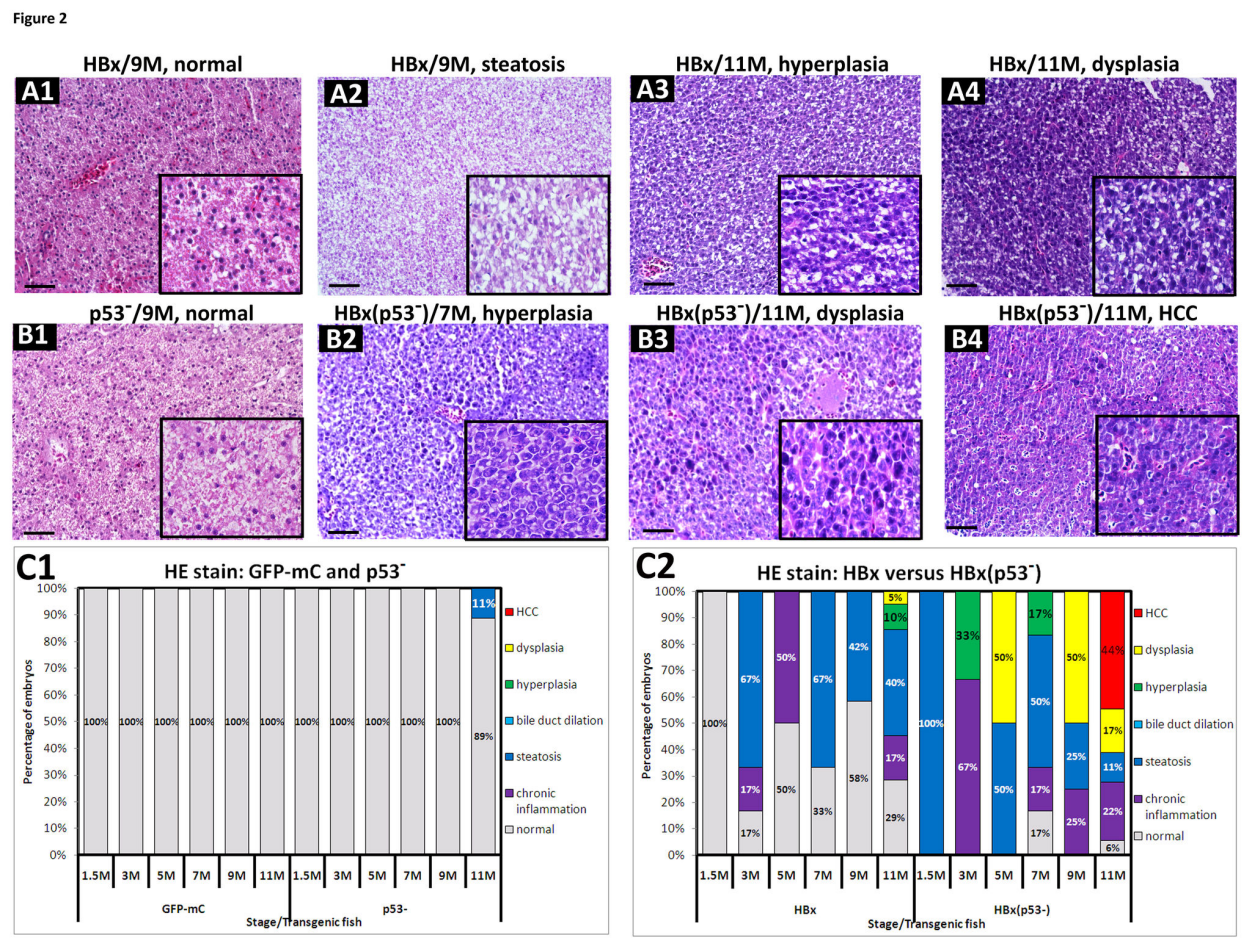
Histopathology of hepatocytes overexpressing HBx in wild-type and mutant p53 transgenic fish at 9 and 11 months of age. (A1~A4) H&E staining of the liver sections from HBx transgenic fish revealed various pathological phenotypes, such as normal tissue, steatosis, hyperplasia and dysplasia. (B1) H&E staining of the liver sections from p53 mutant fish appeared normal. (B2~B4) H&E staining from the liver sections of HBx(p53^-^) transgenic fish revealed hyperplasia, dysplasia and HCC. All specimens are shown under 200-fold magnification. Scale bars: 50 μm. The boxed areas are enlarged images with 400-fold magnification and are shown in the lower right corner. (C1) Statistical analysis of H&E staining results from GFP-mC and p53 mutant control fish. (C2) Statistical analysis of H&E staining results from HBx and HBx(p53^-^) transgenic fish. The different colors denote the different pathological features, as follows: gray-normal, purple-chronic inflammation, blue-steatosis, light blue-bile duct dilation, green-hyperplasia, yellow-dysplasia, and red-HCC, respectively.

According to the criteria mentioned above, at 9 months, some of the HBx transgenic fish appeared normal (Figure 2A1), and others displayed steatosis (Figure 2A2). At 11 months, the livers of the HBx transgenic fish in the wild-type background appeared to have hyperplasia (Figure 2A3) or dysplasia (Figure 2A4). The hepatocytes from p53 mutant fish appeared normal (Figure 2B1). The overexpression of HBx in the p53 mutant background resulted in the earlier and more frequent incidence of hyperplasia and dysplasia (Figure 2B2 and B3). HBx only initiated HCC formation in the p53 mutant background. This condition was characterized by numerous tumor cells with enlarged, polymorphic nuclei and prominent nucleoli (Figure 2B4). In summary, the H&E staining results showed that both the GFP-mC transgenic and the p53 mutant fish appeared normal at all stages (Figure 2C1), while HBx-overexpressing wild-type fish developed low incidences of hyperplasia (10%) and dysplasia (5%) at 11 months. Furthermore, 33% of HBx(p53^-^) fish developed hyperplasia as early as 3 months, and 50% of HBx(p53^-^) fish developed dysplasia at 5 and 9 months. Finally, 44% of HBx(p53^-^) fish developed HCC at 11 months (Figure 2C2). 

### Early pathological alterations in fibrosis, glycogen accumulation, apoptosis and PCNA staining in the livers of HBx transgenic, p53 mutant fish

**Figure 3:**
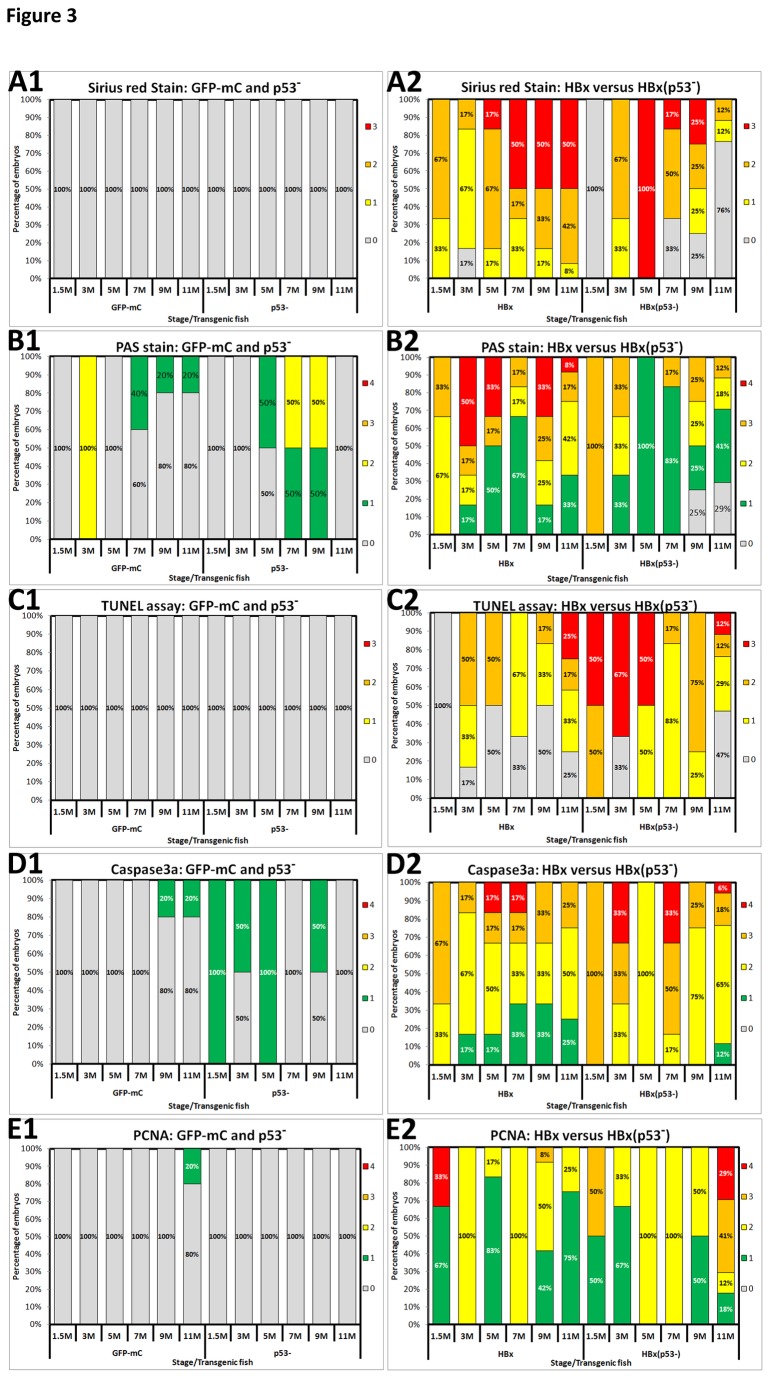
Comparison of the histopathology of hepatocytes among wild-type and p53 mutant fish overexpressing HBx or src from 1.5 to 11 months of age. (A) Liver fibrosis was determined by Sirius Red staining. (B) Glycogen accumulation was identified by periodic acid-Schiff (PAS) staining. (C) Apoptosis was examined using the TUNEL assay. (D) Activated caspase 3a was detected by IHC staining. (E) Nuclear PCNA expression was assessed using IHC staining. For each figure, panel 1 represents GFP-mC and p53 mutant control fish, and panel 2 represents HBx and HBx(p53^-^) transgenic fish. The different colors denote different scores. For A and C, there are four scores in total, as follows: gray-0, yellow-1, orange-2, and red-3. For B, D and E, there are five scores in total, as follows: gray-0, green-1, yellow-2, orange-3, and red-4.

We previously found that the liver-specific overexpression of HBx changes the amount of collagen fibers, glycogen, apoptosis, and cell proliferation [11]. In this study, we compared these pathological changes in HBx transgenic fish in wild-type and p53 mutant backgrounds (Figure S3). In contrast to control fish, which had very low amounts of collagen fibers (Figure 3A1), HBx expression in wild-type zebrafish resulted in high levels of fibrosis in fish ranging in age from 1.5 months to 11 months (Figure 3A2). On the other hand, overexpressing HBx in p53 mutant fish (HBx(p53^-^)-TG2) resulted in the development of fibrosis at 3 months of age, and the amount of collagen fibers decreased as hepatocarcinogenesis progressed (Figure 3A2). In wild-type and p53 mutant fish, the accumulation of glycogen was low to moderate (Figure 3B1), and higher levels were detected in HBx transgenic fish in the wild-type background (Figure 3B2). In the mutant p53 fish expressing HBx, glycogen began to accumulate in hepatocytes as early as 1.5 months of age but decreased with the progression of hepatocarcinogenesis (Figure 3B2). A synergistic effect between p53 mutation and HBx overexpression was observed with respect to apoptosis and activated caspase 3a (Figure 3C and 3D). Although cellular proliferation, as determined by PCNA staining, was detected in HBx(WT) transgenic fish, the highest levels of nuclear PCNA accumulation were observed in the HBx(p53^-^) transgenic fish at 11 months (Figure 3E & Figure S4). The early pathological alterations reflect the advancing HCC progression in the HBx transgenic zebrafish on a p53 mutant background. All the raw data for the staining results are provided in Table S2.

### The up-regulation of lipogenic factors, fibrosis markers, cell cycle-related genes, tumor markers and metastasis-associated genes in HBx transgenic, p53 mutant fish

**Figure 4:**
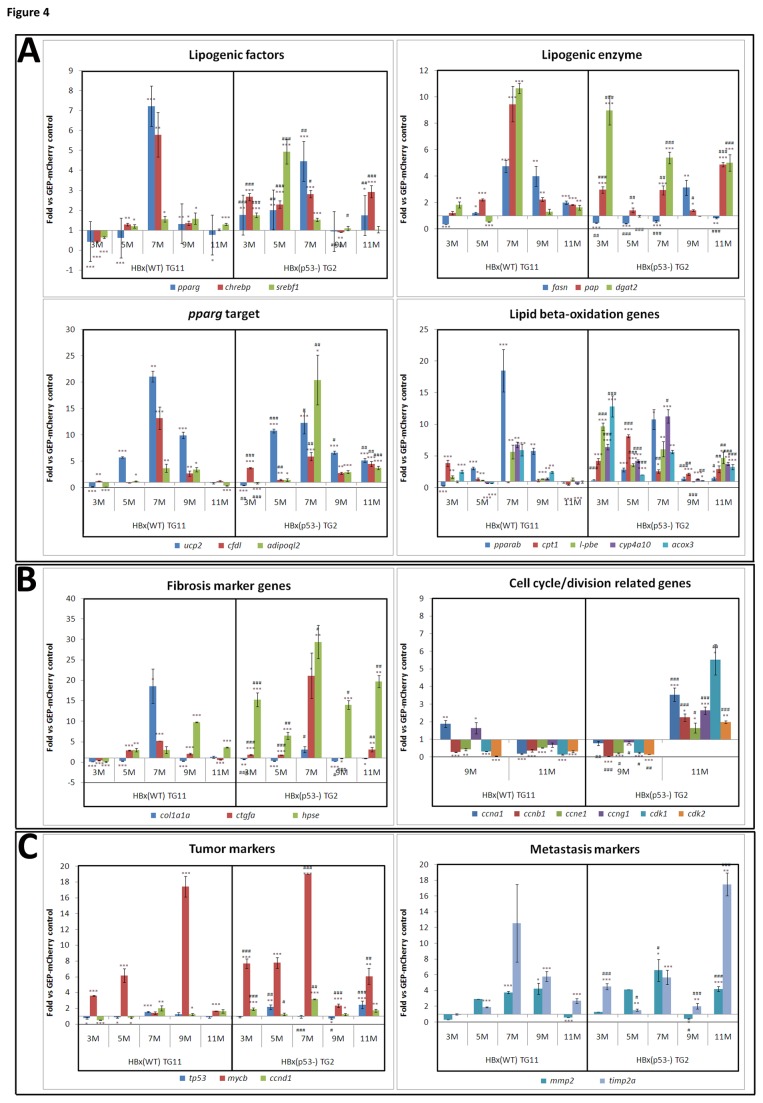
Quantitative RT-PCR analyses of selected marker genes in HBx transgenic fish of the wild-type and p53 mutant backgrounds. (A) Lipid metabolism-associated genes, including lipogenic factors, lipogenic enzymes, PPAR-gamma targets, and lipid beta-oxidation; (B) fibrosis markers and cell cycle/division-associated genes; and (C) tumor markers and metastasis markers were analyzed by Q-PCR. RNAs from the liver samples at five different ages (3, 5, 7, 9, and 11 months) in the Tg(*l-fabp*:HBx-mCherry)(TG11) and Tg(*l-fabp*:HBx-mCherry;*cmcl2*:GFP) transgenic fish (TG2) were analyzed. Each Ct value was normalized using β-actin and was then compared with the Ct value for the GFP-mCherry fish. The delta-delta Ct values were converted into fold differences. Multiple replicates were performed, and the mean values are shown with standard deviations. The differences among the variables were assessed using a two-tailed Student’s *t*-test. The symbol “_*_” represents significance between HBx(WT) and GFP-mCherry control or between HBx(p53^-^) and GFP-mCherry control, and the “#” represents significance between HBx(p53^-^) and HBx(WT). P<0.05 was considered to be statistically significant; _*_, #: 0.01<P≤0.05; _**_, ##: 0.001<P≤0.01; _***,_ ###: P≤0.001.

Using quantitative RT-PCR analysis (Q-PCR), we previously showed that HBx overexpression in wild-type fish can enhance the expression of genes related to the observed pathological changes [11]. We further compared the gene expression profile from wild-type (HBx-TG11) versus p53 mutant (HBx(p53^-^)-TG2) fish overexpressing HBx (Figure 4). As described earlier, the up-regulation of genes involved in lipid metabolism, lipogenic factors and enzymes, PPAR-gamma target genes and lipid beta-oxidation were detected starting at 3 months of age, and their expression levels peaked at 7 months in the HBx(WT) transgenic fish. The expression of the genes related to lipid metabolism was much more profound in the mutant p53 fish overexpressing HBx (Figure 4A). In the HBx(WT) fish, fibrosis markers were up-regulated at 5 to 7 months of age. Moreover, each fibrosis marker tested, *col1a1α*, *ctgfa*, and *hpse*, was expressed at higher levels in the HBx(p53^-^) fish (Figure 4B). Cell cycle/division-related genes were dramatically up-regulated in the HBx(p53^-^) fish at 11 months, but not in the HBx(WT) transgenic fish (Figure 4B). Additionally, the tumor markers *tp53*, *cmycb* and *ccnd1* and the metastasis-associated proteins *mmp2* and *timp2a* were also significantly up-regulated in the HBx(p53^-^) fish compared with the HBx(WT) fish at 11 months (Figure 4C). Taken together, these results demonstrated a synergistic effect between HBx and mutant p53 on the regulation of genes related to cell cycle/division, tumor progression and metastasis.

### The differential activation of src pathways in the HBx transgenic, p53 mutant zebrafish progressed to hyperplasia and HCC

We previously reported the up-regulation of *Src* mRNA in the HBx-induced HCC transgenic mouse model using a systems biology approach [16]. Therefore, we hypothesized that the up-regulation of *src* mRNA may occur during hyperplasia and HCC development in human HCC patients and HBx(p53^-^) transgenic fish. We first examined the expression levels of *SRC* mRNA in HCC patients. We detected *SRC* mRNA up-regulation in a high proportion of HCC patients, specifically, in approximately 60-65% of the 51 HBV-positive HCC patients at three different stages, in approximately 40% of the patients at stages I and II and in up to 67% of patients at stage III of 49 HCV-positive HCC patients (Figure S5A). These results are in agreement with a previous report [28]. Additionally, we examined whether SRC protein overexpression was also observed in other human cancers. SRC protein overexpression was found in 80 cases of malignant tumors, representing 10 tumor types, including breast, cerebrum, colon, esophagus, kidney, liver, lung, prostate, stomach and uterus (Figures S5B and S5C). Our results indicate that the overexpression of SRC (in both mRNA and protein level) plays a role in HCC formation and in the formation of various human cancers.

**Figure 5:**
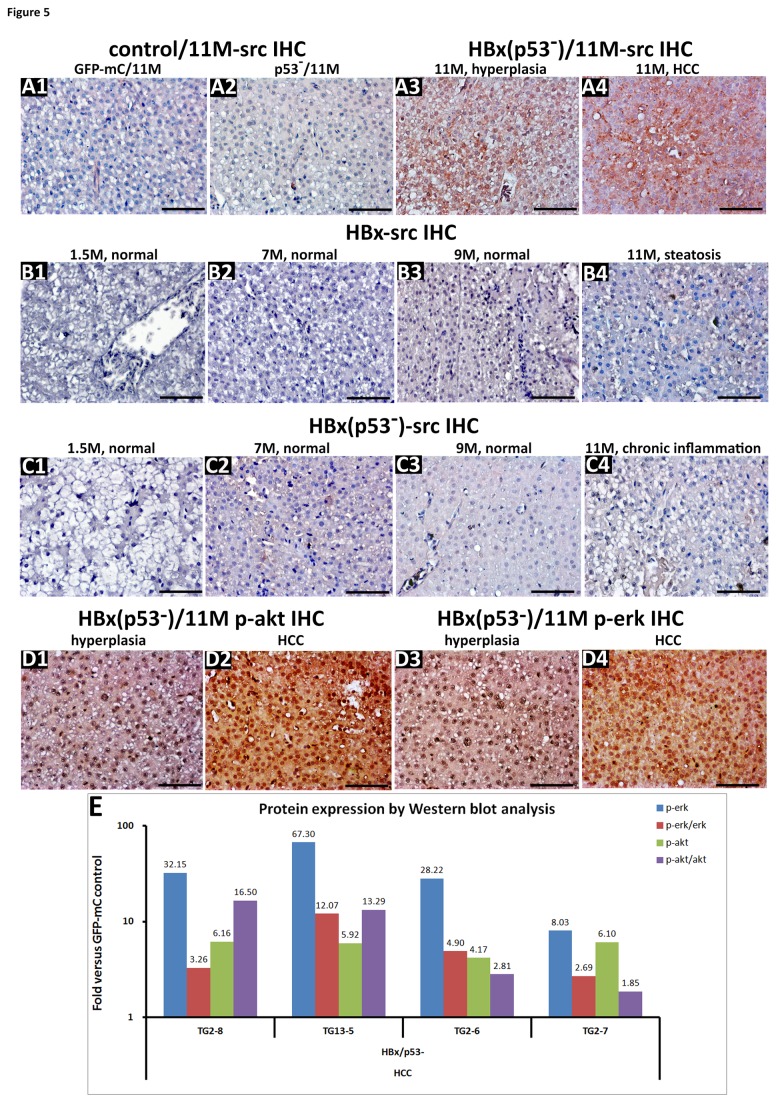
The assessment of *src*, p-ERK and p-AKT signaling in wild-type, p53 mutant and Tg(*l-fabp*:HBx-mCherry;*cmcl2*:GFP) fish with different liver diseases. (A) Immunohistochemical analysis of *src* protein expression in hepatocytes from the GFP-mC and p53 mutant control fish and HBx(p53^-^) transgenic fish at 11 months. (B) src IHC results from HBx transgenic fish at 1.5, 7, 9 and 11 months. (C) src IHC results from HBx(p53^-^) transgenic fish at 1.5, 7, 9 and 11 months. (D) Immunohistochemical detection of phosphorylated ERK and phosphorylated AKT in 11-month-old Tg(*l-fabp*:HBx-mCherry;*cmcl2*:GFP) transgenic fish with hyperplasia and HCC (x 400). Scale bars: 50 μm. (E) Western blot analysis for the activation of erk and akt in the livers of transgenic fish at different stages of HCC development. After measuring the band intensity using the UVP VisionWorks LS software, the relative density was normalized to β–actin. The ratios of p-erk/erk and p-akt/akt were analyzed, and the data are expressed as fold changes of HBx(p53^-^) transgenic fish relative to GFP-mC controls.

Next, we examined src protein expression levels during hyperplasia and HCC in the HBx(p53^-^) transgenic fish by immunostaining. We found that p53 mutant fish expressing GFP-mC had very low expression of src protein (Figure 5A1 and A2). However, src was highly expressed in the livers of HBx(p53^-^) fish that developed hyperplasia and HCC (Figure 5A3 and A4). The src protein was undetectable in the liver specimens from HBx and HBx(p53^-^) fish aged 1.5 to 9 months that appeared normal (Figure 5B1~B3, C1~C3), as well as in the liver sections from fish aged 11 months that had only developed steatosis (Figure 5B4) or chronic inflammation (Figure 5C4). Upon activation, SRC phosphorylates and activates downstream targets, such as AKT and ERK [29]. We found elevated levels of p-akt and p-erk in the HBx(p53^-^) liver during hyperplasia and HCC (Figure 5D) but not in the control fish or the liver specimens from fish that had only developed steatosis (data not shown). Using western blot analysis, we confirmed that the up-regulation of p-akt and p-erk was observed in four HBx(p53^-^) transgenic fish that developed HCC (Figure 5E). Together, these data suggest that the activation of src and its downstream molecules, akt and erk, may contribute to the formation of hyperplasia and HCC in HBx(p53^-^) transgenic zebrafish.

### Generation of liver-specific src transgenic, p53 mutant zebrafish

Because a positive correlation between SRC activation and HCC formation was identified in an earlier report [28] and our experiments, we investigated whether the liver-specific expression of src induces HCC formation and examined the potential synergistic effect between src and p53 mutation. Comparative analysis showed a high level of conservation among the src protein sequences in human, mouse and zebrafish (Figure S6). Therefore, we tested the oncogenicity of zebrafish src by generating the *l-fabp*:src-pA/CG construct, which contains zebrafish src under the control of the *l-fabp* promoter and a GFP reporter under the control of the *cmcl* promoter (Figure S7A). We generated two transgenic lines in the wild-type background Tg(*l-fabp*:src-pA/CG) and three lines in the p53 mutant background Tg(*l-fabp*:src-pA/CG), and transgene expression was demonstrated by visualizing GFP in the hearts of 11-month-old fish (Figure S7B). The expression of the src protein was further verified by immunohistochemistry using an anti-src antibody (Figure S7C). Hepatocytes from src transgenic fish displayed strong expression levels of src protein from 3 to 11 months of age, while the control GFP-mC transgenic fish showed no src expression (Figure S7C). The expression of *src* RNA in five transgenic zebrafish lines was assessed using Q-PCR, and no significant difference was observed between the various lines of src transgenic fish (Figure S7D). The similar protein and mRNA expression levels of src in the src transgenic zebrafish in both the wild-type and p53 mutant backgrounds excludes the possibility that p53 may affect src expression levels. 

### The *Src* transgenic, p53 mutant zebrafish model shows good correlation with *HBx* induced HCC in p53 mutant zebrafish

**Figure 6:**
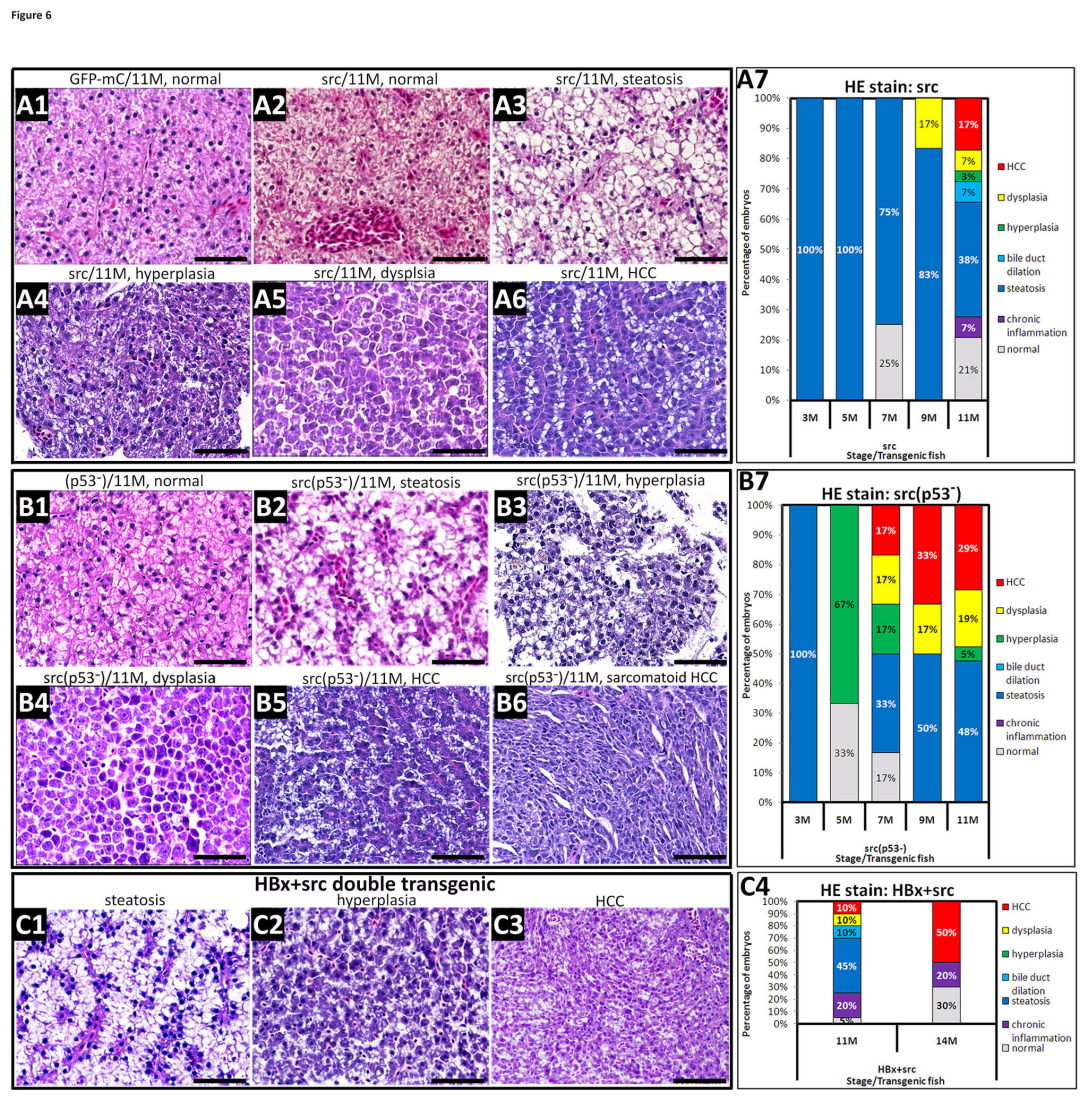
Histopathology of the hepatocytes in *Src*-overexpressing transgenic zebrafish in the wild-type and p53 mutant backgrounds. (A1) H&E staining of liver sections from wild-type fish revealed normal histology at 11 months. (A2~A6) H&E staining of liver sections from *src*-overexpressing, wild-type fish displayed steatosis, hyperplasia, dysplasia and HCC at 11 months. (B1) H&E staining of liver sections from the p53 mutant fish showed normal features at 11 months. (B2~B6) H&E staining of liver sections from src(p53^-^) transgenic fish showed severe steatosis and chronic inflammation, dysplasia, HCC and sarcomatoid HCC at 11 months. (C) The hepatocytes from the double transgenic line overexpressing HBx and src in a wild-type background exhibit steatosis, chronic inflammation, hyperplasia, and HCC. All sections were stained with H&E and photographed at 400X magnification. Scale bars: 50 μm. A7, B7, and C4 show the statistical analysis of the H&E staining results. The following different colors denote the different pathological features: gray-normal, purple-chronic inflammation, blue-steatosis, light blue-bile duct dilation, green-hyperplasia, yellow-dysplasia, and red-HCC.

The liver tissue from src transgenic fish and src(p53^-^) transgenic fish was analyzed, and the detailed results are shown in Table S1. Hepatocytes from the wild-type fish appeared to be normal, even up to 11 months of age (Figure 6A1). The overexpression of *src* was associated with the development of steatosis, hyperplasia, dysplasia and HCC (Figure 6A2 to A6). In the p53 mutant background, the liver appeared normal up to 11 months (Figure 6B1). However, the overexpression of *src* in the p53 mutant fish resulted in the development of steatosis, hyperplasia, dysplasia, and HCC; moreover, sarcomatoid HCC characterized by pleomorphic spindle tumor cells growing in haphazard, fascicular patterns was observed in three out of five cases of HCC (Figure 6B2 to B6). In summary, the H&E results showed that fish overexpressing src in the wild-type background developed a low incidence of hyperplasia (3%) at 11 months, a low incidence of dysplasia at 9 months, and a somewhat higher incidence of HCC (17%) at 11 months (Figure 6A7). Furthermore, 67% of src(p53^-^) transgenic fish developed hyperplasia at 5 months, and 17% developed dysplasia from 7 to 11 months. In addition, HCC was detected at 7 months in approximately 1% of src(p53^-^) fish, and 33% of src(p53^-^) fish developed HCC at 9 and 11 months of age (Figure 6B7). These results suggest that the overexpression of *src* in p53 mutants facilitates the early onset of HCC progression. Furthermore, sarcomatoid HCC was only found in the src(p53^-^) fish, suggesting that there is a synergistic effect between *src* and p53 mutation on hepatocarcinogenesis. 

### Enhanced tumor incidence and onset in double *HBx* and *src* transgenic zebrafish

Because sarcomatoid HCC was only found in the src(p53^-^) fish but not in the HBx(p53^-^) transgenic fish, it is possible that the cause of HBx-induced HCC is different from that of src-induced HCC. To address this question, we crossed Tg(*l-fabp*:HBx-mC) and Tg(*l-fabp*:src-pA/CG) fish to create double transgenic fish expressing HBx and src in a wild-type background. We found that even in the absence of the p53 mutation, 10% of the hepatocytes developed into HCC at 11 months, and 50% of HBx-src double transgenic fish developed HCC at 14 months. Both tumor incidence and onset were enhanced, even in the absence of the p53 mutation (Figure 6C). We concluded that the cause of HBx-induced HCC is similar to that of src-induced HCC and that sarcomatoid HCC is a subtype of HCC that only occurs in src transgenic fish in a wild-type background.

### Activation of p-erk, p-akt, myc, vegf and jnk1 in the liver tumors of the src transgenic, p53 mutant zebrafish

**Figure 7:**
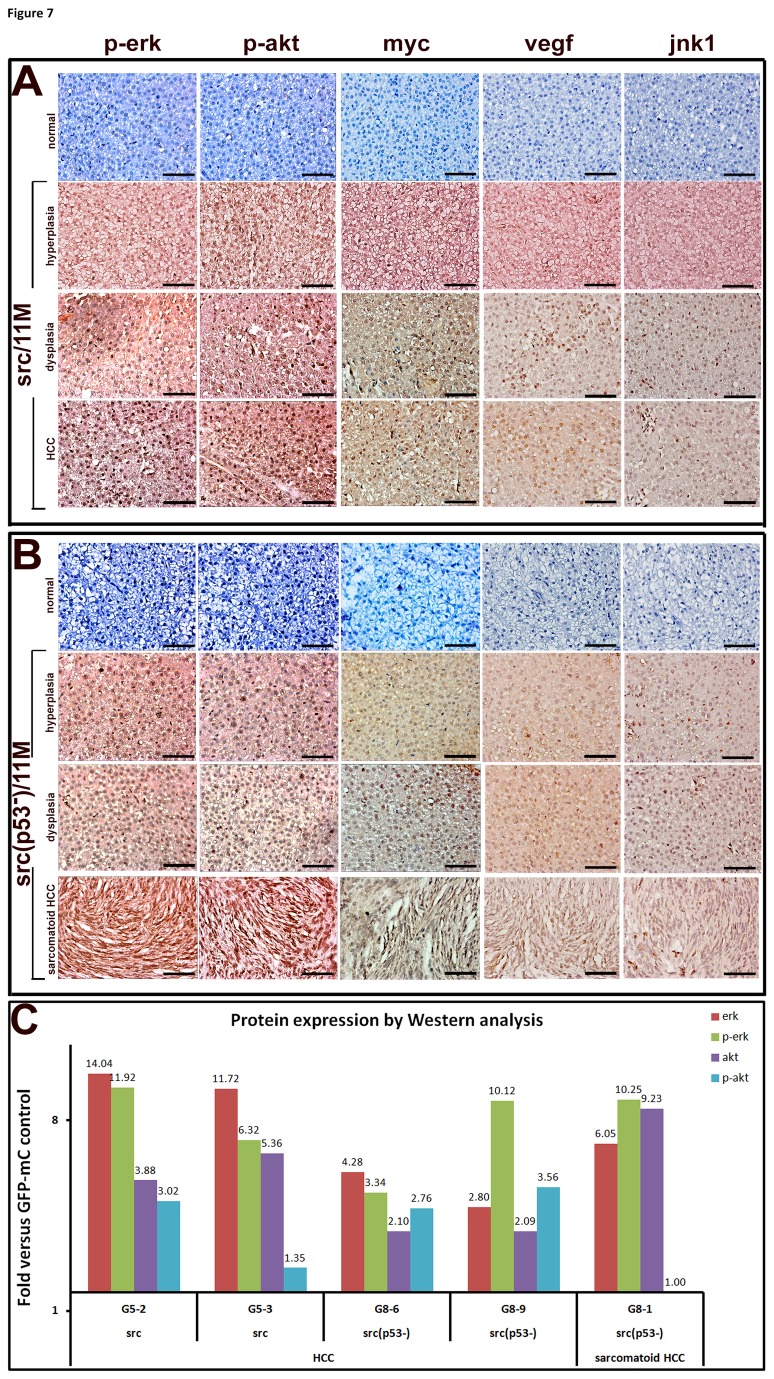
Assessment of p-erk, p-akt, myc, vegf and jnk1 signaling in *Src*-overexpressing wild-type and p53 mutant zebrafish. Immunohistochemical analyses of phosphorylated erk1/2 (p-erk), phosphorylated akt (p-akt), myc, vegf and jnk1 were performed in liver sections prepared from src-overexpressing wild-type zebrafish (A) or p53 mutant zebrafish (B) that displayed hyperplasia, dysplasia, HCC and sarcomatoid HCC (x 400). GFP-mCherry transgenic fish that were 11 months of age were used as controls and are shown in the first row of each panel. Scale bars: 50 μm. (C) Western blot analysis for the activation of erk and akt in the livers of transgenic fish at different stages of HCC development. After measuring the band intensity using the UVP VisionWorks LS software, the relative density was normalized to β–actin. Ratios of p-erk/erk and p-akt/akt were analyzed, and the data are expressed as relative fold changes for src or src(p53^-^) transgenic fish relative to GFP-mC controls.

SRC is linked to the activation of ERK, AKT and JNK signaling pathways in humans. We used an immunohistochemical approach to further evaluate the involvement of these signaling pathways following src overexpression in the zebrafish. In contrast to the exclusively nuclear localization observed in the normal livers of the GFP-mC control transgenic zebrafish (Figure 7A, B, the first rows), strong signals for p-erk, p-akt, myc, vegf and jnk1 were detected in both the nuclei and cytoplasm of the livers in the src-overexpressing transgenic fish (Figure 7A, B) with hyperplasia, dysplasia, HCC and sarcomatoid HCC. We confirmed that p-erk and p-akt were indeed up-regulated in multiple src and src(p53^-^) transgenic fish (Figure 7C). 

**Figure 8:**
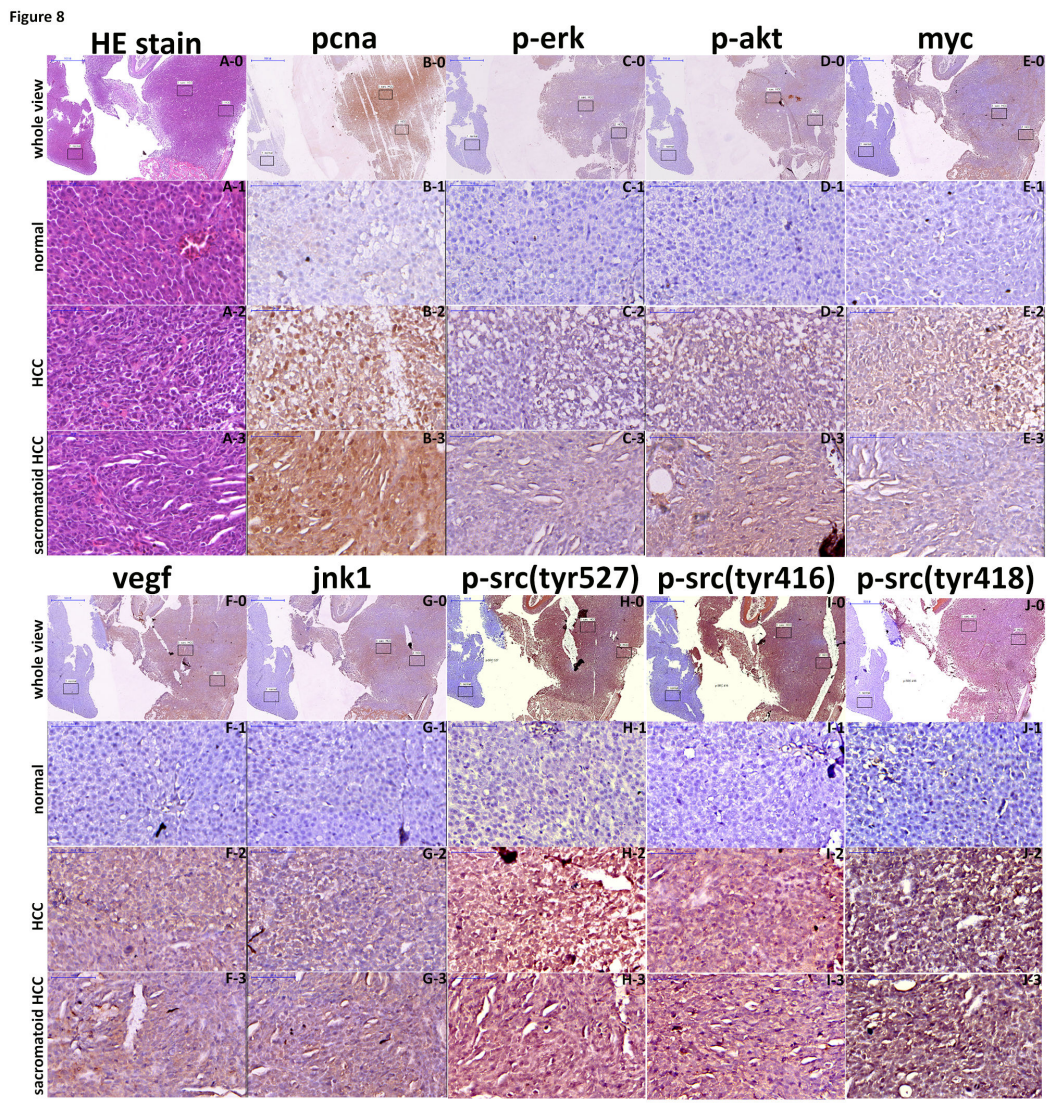
Assessment of pcna, p-erk, p-akt, myc, vegf, jnk1, p-src(tyr527), p-src(tyr416), and p-src(tyr418) signaling in HCC, sarcomatoid HCC, and adjacent normal tissue of src-overexpressing transgenic zebrafish. Liver sections from the src-overexpressing transgenic zebrafish that had developed HCC and sarcomatoid HCC, as well as adjacent normal tissue, were analyzed with H&E staining (A) and immunostaining for pcna, p-erk, p-akt, myc, vegf, jnk1, p-src(tyr527), p-src(tyr416), and p-src(tyr418) (B~J). All slides, comprising normal tissue (1), HCC (2) and sarcomatoid HCC (3), were analyzed using Panoramic Viewer under lower resolution (0) and subsequently at higher resolution. (A0-A3) H&E stain, (B0~B3) pcna protein expression, (C0~C3) p-erk expression level, (D0~D3) p-akt expression level, (E0~E3) myc protein expression, (F0~F3) vegf protein expression, (G0~G3) jnk1 protein expression, (HG0~H3) p-src(tyr527) level, (I0~I3) p-src(tyr416) level, and (J0~J3) p-src(tyr418) expression level were assessed. Scale bars: 50 μm.

Using src/p53 mutant fish, we performed IHC staining on lesions and adjacent normal liver tissues to verify positive staining in the lesions and negative staining in the adjacent normal tissues. In addition to using an antibody against Src, we used an antibody against phosphorylated Src. In Figure 8A, we show the HCC and sarcomatoid HCC nodules together with adjacent normal liver tissue from the same section using H&E staining. Using an antibody against PCNA for IHC, we clearly observed strong staining in HCC and sarcomatoid HCC nodules but very weak staining in the adjacent normal tissues in the same section (Figure 8B). The src downstream proteins, such as p-erk, p-akt, myc, vegf and jnk1, all showed strong staining in HCC and sarcomatoid HCC but very low staining in the adjacent normal tissues (Figure 8C~G). The three phospho-src antibodies used for IHC demonstrated not only that src was overexpressed at the RNA and protein level but also that the phosphorylation of src protein was elevated in HCC and sarcomatoid HCC but very low in the adjacent normal tissues (Figure 8H~J). We verified the positive staining in HCC and sarcomatoid HCC lesions and the negative staining in adjacent normal tissue. Although the normal tissue and HCC lesions were in the same section, they were from different parts of the liver and, as such, could only be observed under lower resolution. In previous work showing that inducible Xmrk caused a high incidence and early onset of HCC in transgenic zebrafish [30], the authors also found that the HCC induced in the transgenic fish was quite uniform and that the upregulation of p-ERK and p-STAT5 occurred uniformly throughout the sections. Another study showed that the overexpression of KrasV12 induced malignant tumors in zebrafish at 9 months, and histological analysis confirmed that the tumor was of HCC grade II-III and that the histopathology of HCC was highly uniform [31]. In both studies, the authors used the same promoter (l-fabp) as was used in our study. It is possible that the l-fabp promoter drove uniformly high expression of the transgenes and transformed the entire tissue to tumor tissue.

### The penetrance of HBx and src in the p53 mutant background induced pathological changes

**Figure 9:**
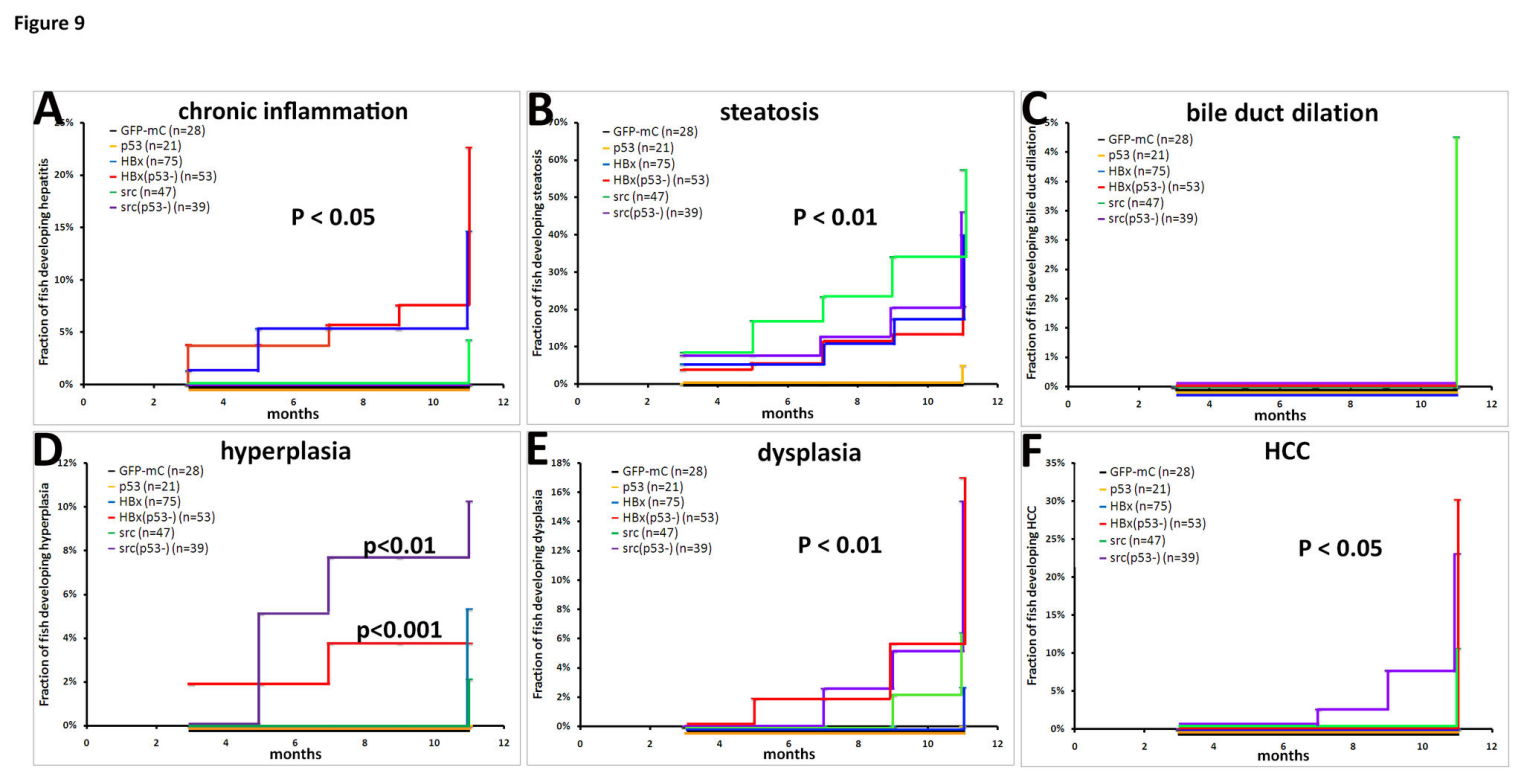
Cumulative frequency showing the fraction of fish that had developed the various liver diseases. The cumulative frequency of transgenic fish from multiple lines with chronic inflammation (A), steatosis (B), bile duct dilation (C), hyperplasia (D), dysplasia (E) and HCC (F) were analyzed by Kaplan-Meier analysis. The following different colors denote the different fish lines: black-GFP-mC control, orange-p53 mutant, blue-HBx transgenic fish in wild-type background, red-HBx(p53^-^) transgenic fish, green-src transgenic fish in wild-type background, and purple-src(p53^-^) transgenic fish. P<0.05 was considered to be statistically significant.

We compared the four following transgenic fish, HBx, HBx(p53^-^), src, and src(p53^-^), with GFP-mC and p53 mutant control fish by Kaplan-Meier analysis (Figure 9). The results showed that HBx(p53^-^) and src(p53^-^) significantly (P<0.01) induced hyperplasia and dysplasia compared to control fish. Furthermore, only HBx(p53^-^), src, and src(p53^-^) fish developed HCC (P<0.05). Earlier phenotypic changes, such as chronic inflammation or steatosis, occurred at significantly different rates in the HBx and src transgenic fish (P<0.05 and P<0.01, respectively). 

## Discussion

Hepatocarcinogenesis is initiated from steatosis, inflammation, fibrosis, and cirrhosis, and finally develops into HCC. In Asia, more than 80% of all HCC cases are the result of infection with either HBV or HCV [32]. Overall, virus-induced HCC patients have pre-existing cirrhotic conditions, and the contribution of fibrosis-dependent mechanisms to HCC has been reviewed extensively [29]. HBx-induced hepatocarcinogenesis is associated with chromosomal alterations and mutations [33] and the activation of specific oncogenic pathways [34,35]. In this report, we have established a zebrafish HCC model using HBx and src overexpression in a p53 mutant background and revealed the synergistic effect of the most relevant risk factors for human HCC. 

HBx transgenic mice show increased hepatic lipid accumulation [36], which often develops into large liver cell dysplasia [37]. Dysplasia can then evolve into HCC, and this process often occurs together with c-myc [38] or AFB1 [39], although HBx itself can also induce HCC [16,40,41]. However, in zebrafish, it appears to be more difficult for HBx alone to induce tumor formation. It was reported that HBx can induce increased hepatic fat accumulation [9,11] and, when combined with hepatitis C virus core protein, can induce the development of cholangiocarcinoma [42]. There have been no published reports that HBx, alone or combined other oncogenic factors, can induce HCC in zebrafish. The novelty of our report is that we combined HBx with p53 mutant zebrafish and found that these transgenic zebrafish progressed to HCC.

The synergistic effect between HBx and p53 has not previously been reported. The p53 protein affects the stability of the HBx protein [43], and p53-mediated HBx degradation is MDM2-dependent [44]. The direct interaction between HBx and p53(249mutant) protein confers early growth advantages [45]. In tumor cell lines, HBx-induced p53 phosphorylation through ATM kinase has been shown to have a pro-apoptotic effect; however, it has opposite effects in non-tumor cells [46]. Furthermore, HBx can modulate p53 transcription regulation by altering the recruitment of p53-associated transcription cofactors and coregulators [47]. However, these results were obtained in vitro using cell culture models, whereas animal models for the synergistic effect between HBx and mutant p53 were lacking.

From a study of HCC in West Africa, it was found that complete HBx sequences are often associated with the presence of the TP53 R249S mutation [48]. From a case-control study in Thailand, it was found that HBx was associated with the p53(R249S) mutation in HCC in patients with no documented prior cirrhosis [49]. It was also reported that HBV-related tumors have a higher rate of p53 inactivation by mutations [50]. Those reports indicate that the synergism between HBx and mutant p53 in HCC formation might also occur in human patients.

The activation of Src by HBx during the development of HCC has been reported by several groups and suggests that HCC arising from HBx overexpression in this compound transgenic fish may occur via Src. HBx is able to disrupt intercellular adhesion in a src-dependent manner [51] and can activate the wnt/beta-catenin pathway by suppressing glycogen synthase kinase 3 activity via the activation of Src kinase [52]. HBx has also been reported to activate src kinase, phosphorylate the Raf-1 protein, and stimulate Raf-1 mitochondrial translocation [53]. With regards to the male predominance of HCC, HBx-mediated activation of the c-Src kinase has been shown to result in the phosphorylation of the androgen receptor (AR) and to increase AR-mediated transcriptional activity [54]. Previously, we found the up-regulation of Src mRNA in the HBx-induced HCC mouse model [16]. In this study, we demonstrated that the up-regulation of SRC is common in HCC and in other human cancers. We also demonstrated that HBx overexpression in p53 mutant fish facilitates HCC progression, which has been associated with the up-regulation of *src*, the increased phosphorylation of the src protein, and the activation of downstream signaling pathways. HBx can enhance the expression of Src and can also activate Src by phosphorylation. Additional Src expression can enhance the activation of downstream pathways and facilitate HCC formation. 

In the HBx-induced HCC mouse model in which HBx was driven by the albumin promoter, all of the four lines of HBx transgenic mouse developed HCC at approximately 14 to 18 months [16]. In another HBx transgenic mouse model, large liver cell dysplasia developed at 3 months; this condition progressed to nodules of hepatocellular adenoma and, eventually develops into HCC [37]. The double transgenic mouse model coexpressing HBx (amino acids 58-154) and the murine c-myc gene developed HCC after a prolonged latent period [38]. In all of the HBx mouse models, there was no cirrhosis or fibrosis before HCC formation. Mechanistically, the mouse model does not mimic human HCC because hepatocarcinogenesis in humans progresses gradually from steatosis to fibrosis, hyperplasia and dysplasia, before finally developing into HCC. Our HBx-induced HCC zebrafish model is more similar to human HCC, as the animals progress from steatosis to fibrosis, hyperplasia and dysplasia, prior to developing HCC. Our zebrafish model also shares similar molecular mechanisms with human hepatocarcinogenesis in terms of the activation of src and its downstream signaling pathways. Cancer is a complex disease, and studying the synergy between various oncogenic factors is important for understanding human cancer formation. 

Synergy between oncogenic factors in tumor formation has been reported in zebrafish. Whereas the overexpression of MYCN resulted in the development of neuroblastomas with a penetrance of less than 20%, its coexpression with the activated ALK receptor tyrosine kinase markedly enhanced both the onset and penetrance of the disease [21] The overexpression of KrasV12 induced liver tumor with a penetrance of 28% in p53^+/+^ fish, which increased to 32% in p53^-/-^ fish [31]. The overexpression of a BRAF mutant form (V600E) under the control of the melanocyte mitfa promoter causes melanoma, and activated BRAF cooperates with p53 mutations, resulting in the more rapid development of invasive melanomas [55]. In another study involving melanomas, C-MYC overexpression was found to be required for the continuous suppression of BRAF(V600E) or NRAS(Q61R)-induced senescence [56]. As such, it is common to observe low penetrance with the overexpression of a single oncogene, and the combination with an additional oncogenic factor can enhance its oncogenic activity. Zebrafish is a great model system for testing the synergy between various oncogenic factors.

The tumor suppressor gene p53 is frequently mutated in human cancers, such as HCC [5]. TP53 gene mutations occur in 30% to 55% of hepatocellular carcinomas, and a specific mutation at codon 249 (AGG-->AGT) was shown to correlate with AFB1 contamination [57]. Some p53 mutations result in a loss of tumor suppressor function, while others result in a gain of oncogenic activity [58]. In a study of Chinese HCC patients, 37.2% of patients displayed p53 mutations, and this characteristic was correlated with a shorter average survival. The tumor cells of these patients also exhibited stem cell-like properties [59]. Two of the mutant p53 zebrafish lines were viable and exhibited mutations similar to those found in human cancers (p53(N168K) and p53(M214K)) [55]. It has been shown that a p53 mutation together with the BRAF mutation causes invasive melanomas [55], while an overexpression human oncogenic NRAS(Q61K) mutant in a p53 mutant background led to the development of melanoma [60]. In this study, we used p53(M214K) fish to generate HBx and src transgenic fish and found that the p53 mutation, together with either HBx or src, induced the development of HCC, while the p53(M214K) single mutant fish did not develop HCC. The specific mechanism by which p53 mutations interact with HBx and src to promote cancer development is currently under investigation.

Src kinase is closely associated with cancer progression and metastasis in humans. The enhanced activity of Src kinase correlates positively with cell proliferation, angiogenesis, motility, invasion and migration [61]. The activation of SRC is strongly correlated with the early stages of HCC development [62]; however, the exact function of SRC in the development of liver tumors remains elusive. Using the HBx transgenic zebrafish model, we found that the overexpression of src occurred exclusively in the liver specimens exhibiting hyperplasia and HCC but not in specimens exhibiting chronic inflammation or steatosis. The upregulation of src in our HBx-p53 mutant transgenic fish during HCC formation is similar to a previously reported rat model, in which the kinase activity of pp60c-src was higher in poorly differentiated HCC [63], and to human HCC, in which activated c-Src is observed in the early stages of the disease [62]. The overexpression of HBx in p53 mutant fish resulted in the development of HCC in 44% of the fish at 11 months. The overexpression of src in the p53 mutant fish caused the early onset of tumor formation. The expression levels of src mRNA in the src(p53^-^) fish were 200 times higher than that in the HBx(p53^-^) fish (data not shown). The expression levels of src induced by HBx were significantly lower than in the src transgenic fish. The discrepancy in the expression levels of src might explain the differences in the incidence and rate of tumor formation between HBx(p53^-^) and src(p53^-^) fish. 

Src regulates cell behavior through the regulation of numerous signal transduction pathways [64]. Many different factors can enhance Src activity, including the loss of regulation at its inhibitory C-terminal tyrosine residue due to reduced C-terminal Src kinase (CSK) levels [65]. Src exerts transforming effects via the activation of its downstream signaling pathways, including the phosphatidyl inositol 3-kinase (PI3K), mitogen-activated protein kinase (MAPK) and signal transducer and activator of transcription 3 (STAT3) pathways [66]. HBx induces HCC development by the activation of SRC kinase and the downstream Ras-Raf-MAP kinase pathway [67] and can disrupt adherens junctions in a src-dependent manner [51]. Activated c-Src in HCC may also contribute to resistance against the apoptotic and/or anti proliferative properties of TGF-beta1 [68]. 

Our findings suggest that the possible common mechanism by which p53 and HBx induce hepatocarcinogenesis in lower vertebrates as well as in mammals is through the activation of src and its downstream genes. This study showed that the p53 mutation and HBx synergistically contribute to HCC formation. Our HBx-induced HCC mouse model provided evidence that HBx can induce the expression of Src RNA. In this study, we found that the overexpression of src can increase the p-erk, p-akt, myc, vegf and jnk1 protein levels in src transgenic fish. It is likely that the signaling pathways that are activated during hepatocarcinogenesis are activated by src. This complicated interaction explains the synergism between HBx, src, and p53 mutation. Currently, we are generating multiple transgenic fish lines that express all three oncogenes to increase the penetrance of the HCC formation for drug screening purposes. The results from our animal model study demonstrate another possible mechanism for HBx-induced HCC development, in which the up-regulation of SRC and the activation of its downstream pathways induce oncogenic transformation.

The previous studies using transgenic zebrafish supports the idea of using zebrafish as a potential in vivo platform for drug screening. In an inducible *krasV12* transgenic zebrafish model [15] with a high incidence of HCC formation, liver tumorigenesis has been shown to be suppressed by the inhibition of the Raf-MEK-ERK and PI3K-AKT-mTOR pathways using PD98059, LY294002, and rapamycin. PD98059 has been demonstrated to inhibit human HCC [69], LY294002 has been shown to decrease the capacity of HCC cell migration and invasion of liver tumors [70], and rapamycin treatment has been shown to significantly delay hepatocarcinogenesis in AKT/Ras mice [71]. In another paper, inducible Xmrk, a naturally occurring mutated form of the EGFR isoform EGFRb, was shown to produce a high incidence and early onset of HCC in transgenic zebrafish [30], and experiments with the inhibitors PD98059 (MEK/Erk inhibitor) and nicotinohydrazide (Stat 5 inhibitor) demonstrated that this model was useful for drug screening. The Stat 5 inhibitor was shown to decrease the HBx-dependent activation of EMT-related protein expression and cell mobility in a liver cancer cell line. The use of inhibitors for Raf/MEK/ERK, PI3K/Akt/mTOR, and Jak/STAT signaling in tyrosine kinase receptors (TKR)-based systemic therapy for HCC was recently published [72]. It has been shown that zebrafish HCC displays the same drug response as human HCC. As such, our zebrafish HCC model involving HBx and src overexpression and the p53 mutant background, both mimics human HCC formation and provides potential *in vivo* platforms for drug screening to find therapies for human liver cancer.

## Supporting Information

Figure S1 **Typical histological features of liver of HBx and src transgenic fish (**B**-**H**) in comparison with these of GFP-mCherry transgenic fish.** (A) Liver of GFP-mCherry transgenic fish. Normal liver tissue cells arranged in neat rows, the size of the nucleus are similar, and the nuclear-cytoplasmic ratio is not too high. (B) Cholestasis: deposition yellow-green globular bilirubin pigment, arrows indicated bile and spongy vacuoles. (X200). (C) Steatosis: prominent vacuoles in the cytoplasm of hepatocytes, arrows indicated lipid droplets formed by the vacuoles. (X200). Hyperplasia and dysplasia was based on the degree of cell proliferation, also consider the whole area overall differentiation and cell differentiation, such as nuclear-cytoplasmic ratio etc. (D) Hyperplasia: disordered proliferation of atypical hepatocytes with enlarged and mildly irregular nuclei, arrows indicated single or several larger cells with higher nuclear-cytoplasmic ratio compared to surrounding adjacent normal cells (X200). (E) Dysplasia: transformed cells with enlarged nuclei and prominent nucleoli. (X200). Hepatoma cell morphology differs in texture due to the degree of differentiation, such as poorly differentiated, well differentiated, moderately differentiated etc. (F) Hepatocellular Carcinoma (HCC): hepatocellular carcinoma with marked cystic degeneration (spongiosis hepatis) (X200). (G) Hepatocellular Carcinoma (HCC): severe sheets of tumor cells with enlargement polymorphic nuclei and prominent nucleoli. (X200). (H) Sarcomatoid HCC: pleomorphic spindle tumor cells growing in haphazardly fascicular patterns. (X200).
(TIF)

Figure S2 **Oil-red staining proved the prominent vacuoles in the cytoplasm of hepatocytes of transgenic fish exhibited lipid accumulation.** (A~C) The liver samples from GFP-mC control fish and two strains of HBx transgenic fish in p53 mutant background were stained with hematoxylin-eosin after paraffin embedding. (D~E) The same liver were frozen and stained with oil red O indicated the lipid accumulated in the vacuoles in the hepatocytes. (x 400). Scale bars: 50μm.
(TIF)

Figure S3 **Scoring standard various staining.** (A) Sirius Red stain (x 200), (B) TUNEL assay (x 200), (C) PAS staining (x 200), (D) caspase 3 stain (x 400) and (E) nuclear PCNA stain (x 400). Scale bars: 50μm.
(TIF)

Figure S4 **Representative images of PCNA staining in HBx overexpression transgenic fish.** (A) EGFP-mCherry transgenic fish, (B) HBx overexpression in wild-type, or (C) HBx overexpression in p53 mutant transgenic fish stained with PCNA antibody 7, 9 and 11 months (x 400).Scale bars: 50μm.
(TIF)

Figure S5 **Expression of *SRC* mRNA in human HCC samples and SRC protein various normal tumors tissues of human samples.** (A) Src mRNA expression analyzed by quantitative RT-PCR analysis in the stage I to III HBV or HCV positive HCC samples. (B) To assess the Src expression of the various normal and tumors tissues, the staining intensity of IHC were classified into five scores from 0 to 4. Each IHC result was evaluated and given a score and then average the scores pooled from the same stage of disease from the specific staining. Over-expression of SRC was found in breast, cerebrum, colon, esophagus, kidney, liver, lung, prostate, stomach, and uterus tumors. (C) Representative images of SRC staining in various normal and tumors tissues (x 200). Scale bars: 50μm.
(TIF)

Figure S6 **Amino acid sequence alignment of SRC protein sequence from human, mouse, and zebrafish.** Mining the zebrafish genome assembly database (http://www.ncbi.nlm.nih.gov/genbank/) revealed a cDNA sequence encoding a hypothetical 534-aa protein (GenBank accession no. NP_001003837.2). The putative polypeptide sequence shares 83% amino acid homology with human Src (GenBank accession no. NP_005408.1) with a conserved SH3 domain, SH2 domain and Tyrkc domain. The conservation scoring is performed by PRALINE (http://www.ibi.vu.nl/programs/pralinewww/). The scoring scheme works from 0 for the least conserved alignment position, up to 10 for the most conserved alignment position. The color assignments are blue represents unconserved to red represents conserved.
(TIF)

Figure S7 **Generation and characterization of Tg (*l-fabp*:src-mC/*CG*) transgenic zebrafish.**
(A) Diagram of the *l-fabp*:src-mC/*CG* construct used in this study, containing Tol2 sequences, and the *cmlc2*:GFP expression cassette. (B) The src protein was expressed in the liver of wild-type and p53 mutant fish carrying the *l-fabp*:src-mC/*CG* transgene, as indicated by the *cmlc2*:GFP was expressed in the heart, as indicated by the green fluorescence. (C) (x 200) Immunohistochemical and (D) quantitative RT-PCR analysis of the expression of the src in hepatocytes from the 3, 5, 7, 9 and 11month old wild-type and p53 mutant fish overexpressing *src* and control *l-fabp*:GFP-mC transgenic fish. Scale bars: 50μm.
(TIF)

Table S1 **Summary of H&E stain revealed the pathology of liver tumor progression in GFP-mCherry, p53 mutant, HBx and *src* transgenic fish in wild-type and p53 mutant background, as well as the HBx+src double transgenic fish.**
(DOCX)

Table S2 **Summary of Sirius red Stain, PAS stain, TUNEL assay, Caspase3a and PCNA IHC results in GFP-mCherry, p53 mutant, HBx and *src* transgenic fish in wild-type and p53 mutant background.**
(DOCX)

Table S3 **Primer sequence.**
(DOCX)

Experimental Procedures S1 **The detail information for material and methods are all described in this supporting information file**
(DOC)
