# Supplementary figures and images for: Liver-Specific Expressions of HBx and src in the p53 Mutant Trigger Hepatocarcinogenesis in Zebrafish
Source: PLoS One. 2013 Oct 9;8(10):e76951. doi: 10.1371/journal.pone.0076951 (PMC3793937; doi:10.1371/journal.pone.0076951)

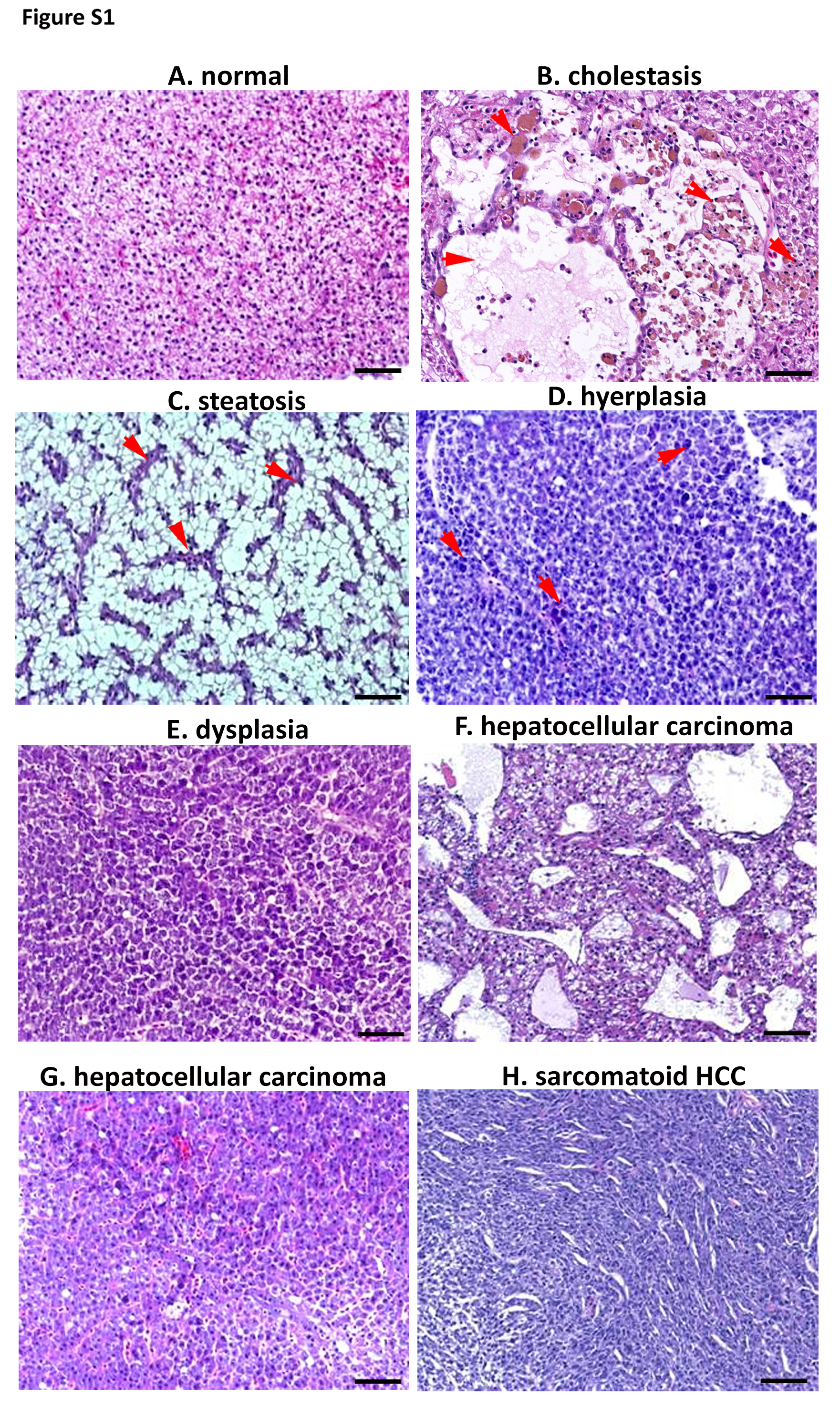

Supplement: Figure S1 — Typical histological features of liver of HBx and src transgenic fish (B-H) in comparison with these of GFP-mCherry transgenic fish. (A) Liver of GFP-mCherry transgenic fish. Normal liver tissue cells arranged in neat rows, the size of the nucleus are similar, and the nuclear-cytoplasmic ratio is not too high. (B) Cholestasis: deposition yellow-green globular bilirubin pigment, arrows indicated bile and spongy vacuoles. (X200). (C) Steatosis: prominent vacuoles in the cytoplasm of hepatocytes, arrows indicated lipid droplets formed by the vacuoles. (X200). Hyperplasia and dysplasia was based on the degree of cell proliferation, also consider the whole area overall differentiation and cell differentiation, such as nuclear-cytoplasmic ratio etc. (D) Hyperplasia: disordered proliferation of atypical hepatocytes with enlarged and mildly irregular nuclei, arrows indicated single or several larger cells with higher nuclear-cytoplasmic ratio compared to surrounding adjacent normal cells (X200). (E) Dysplasia: transformed cells with enlarged nuclei and prominent nucleoli. (X200). Hepatoma cell morphology differs in texture due to the degree of differentiation, such as poorly differentiated, well differentiated, moderately differentiated etc. (F) Hepatocellular Carcinoma (HCC): hepatocellular carcinoma with marked cystic degeneration (spongiosis hepatis) (X200). (G) Hepatocellular Carcinoma (HCC): severe sheets of tumor cells with enlargement polymorphic nuclei and prominent nucleoli. (X200). (H) Sarcomatoid HCC: pleomorphic spindle tumor cells growing in haphazardly fascicular patterns. (X200). (TIF) [file pone.0076951.s001.tif]

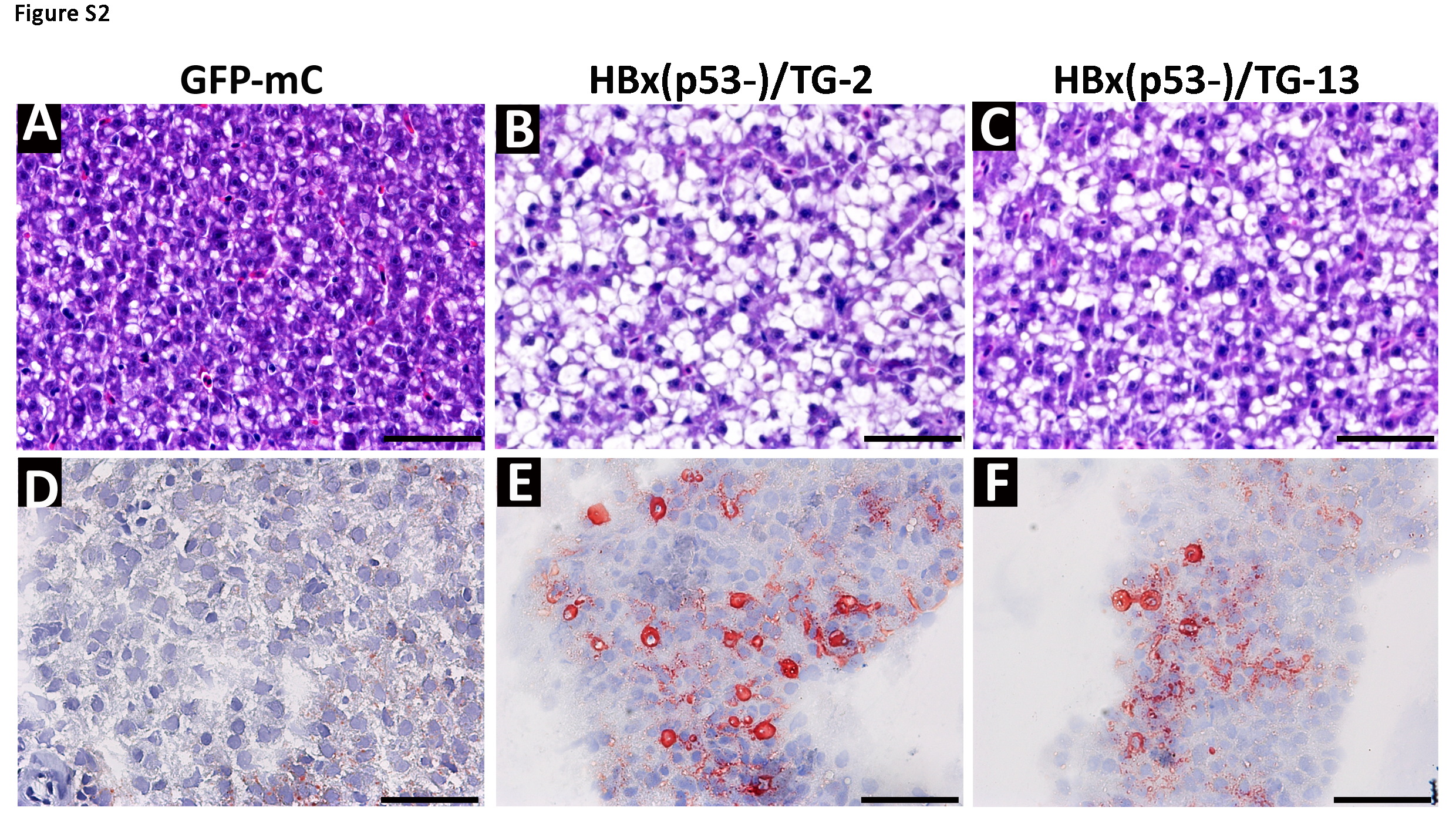

Supplement: Figure S2 — Oil-red staining proved the prominent vacuoles in the cytoplasm of hepatocytes of transgenic fish exhibited lipid accumulation. (A~C) The liver samples from GFP-mC control fish and two strains of HBx transgenic fish in p53 mutant background were stained with hematoxylin-eosin after paraffin embedding. (D~E) The same liver were frozen and stained with oil red O indicated the lipid accumulated in the vacuoles in the hepatocytes. (x 400). Scale bars: 50μm. (TIF) [file pone.0076951.s002.tif]

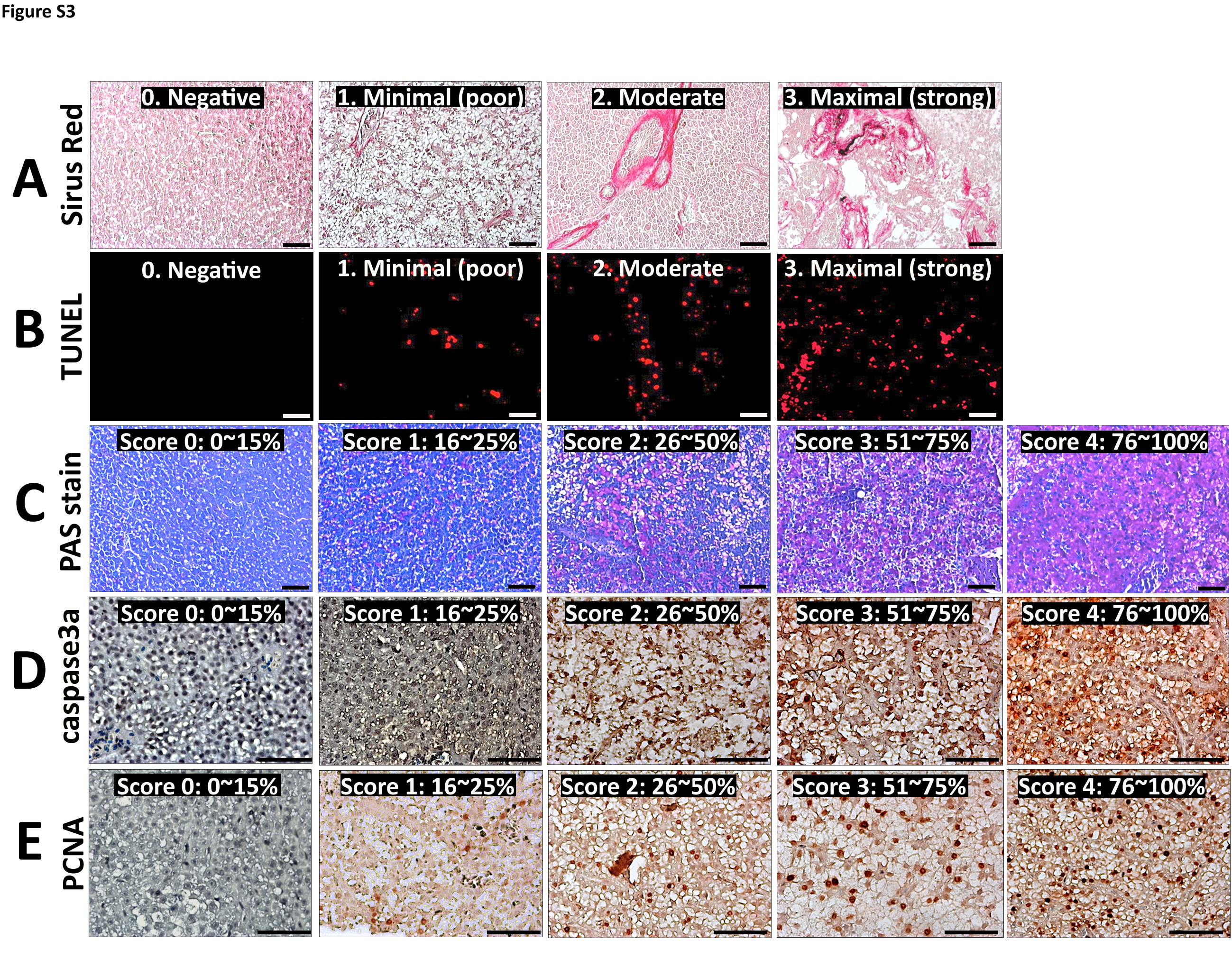

Supplement: Figure S3 — Scoring standard various staining. (A) Sirius Red stain (x 200), (B) TUNEL assay (x 200), (C) PAS staining (x 200), (D) caspase 3 stain (x 400) and (E) nuclear PCNA stain (x 400). Scale bars: 50μm. (TIF) [file pone.0076951.s003.tif]

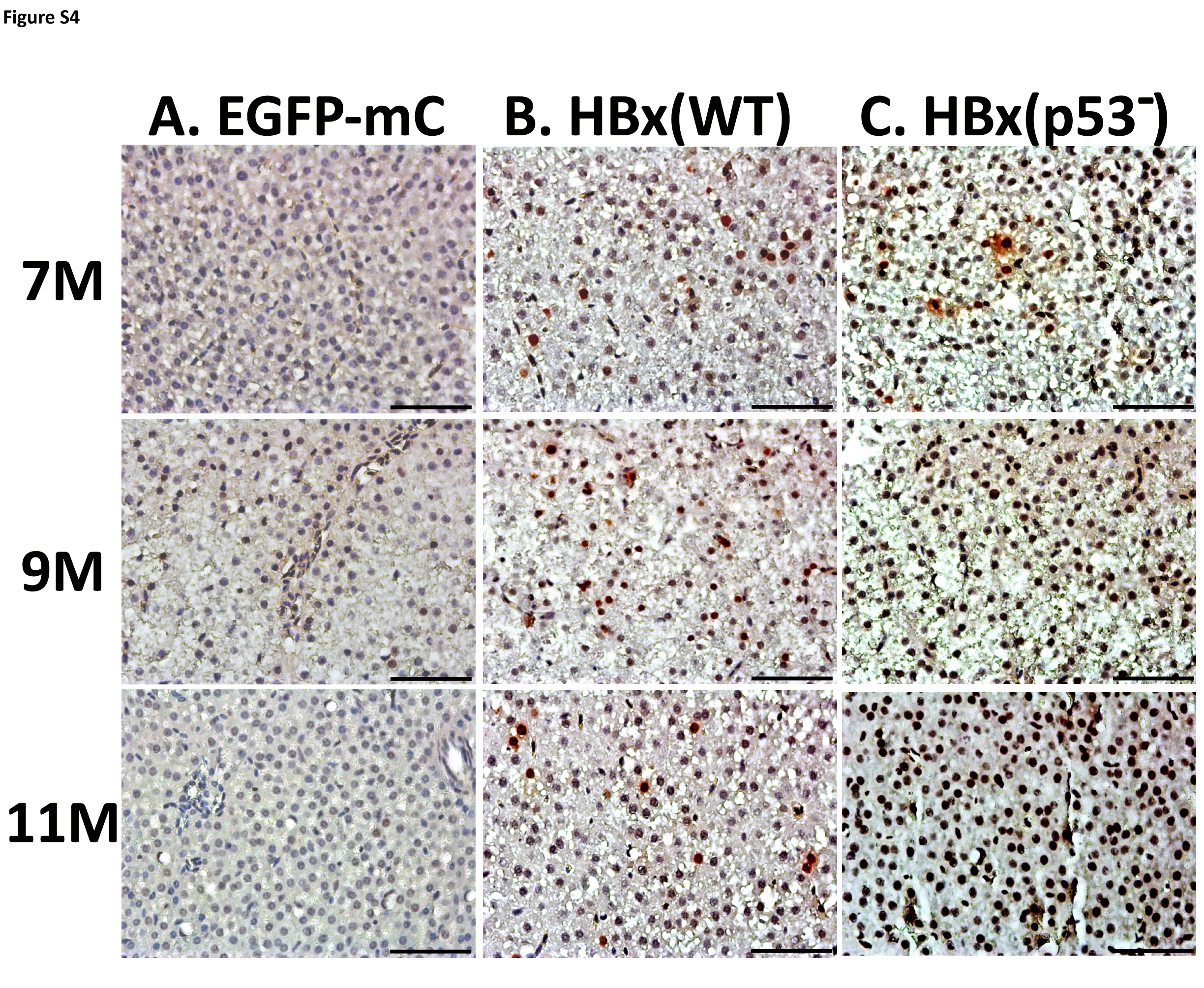

Supplement: Figure S4 — Representative images of PCNA staining in HBx overexpression transgenic fish. (A) EGFP-mCherry transgenic fish, (B) HBx overexpression in wild-type, or (C) HBx overexpression in p53 mutant transgenic fish stained with PCNA antibody 7, 9 and 11 months (x 400).Scale bars: 50μm. (TIF) [file pone.0076951.s004.tif]

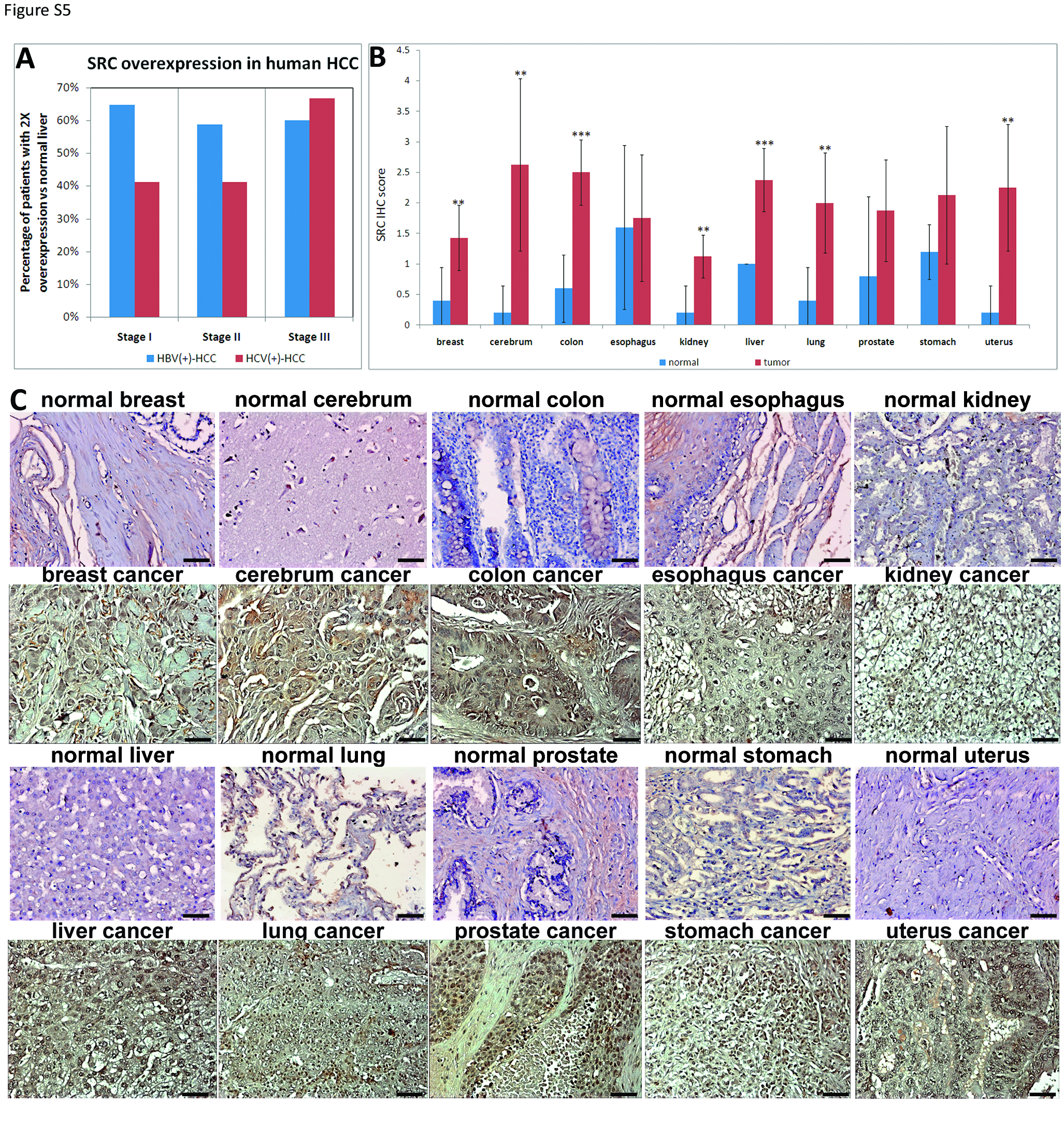

Supplement: Figure S5 — Expression of SRC mRNA in human HCC samples and SRC protein various normal tumors tissues of human samples. (A) Src mRNA expression analyzed by quantitative RT-PCR analysis in the stage I to III HBV or HCV positive HCC samples. (B) To assess the Src expression of the various normal and tumors tissues, the staining intensity of IHC were classified into five scores from 0 to 4. Each IHC result was evaluated and given a score and then average the scores pooled from the same stage of disease from the specific staining. Over-expression of SRC was found in breast, cerebrum, colon, esophagus, kidney, liver, lung, prostate, stomach, and uterus tumors. (C) Representative images of SRC staining in various normal and tumors tissues (x 200). Scale bars: 50μm. (TIF) [file pone.0076951.s005.tif]

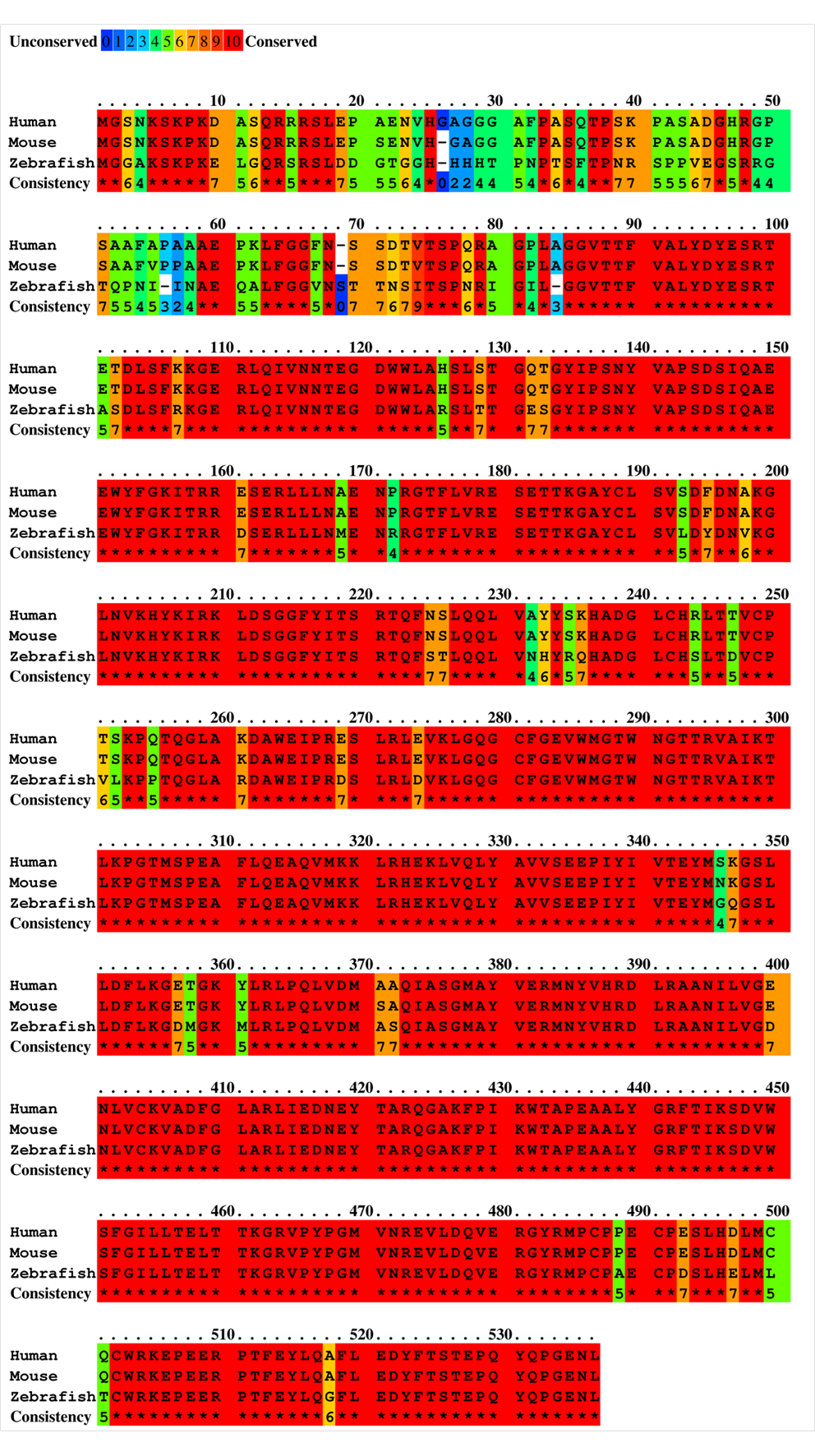

Supplement: Figure S6 — Amino acid sequence alignment of SRC protein sequence from human, mouse, and zebrafish. Mining the zebrafish genome assembly database (http://www.ncbi.nlm.nih.gov/genbank/) revealed a cDNA sequence encoding a hypothetical 534-aa protein (GenBank accession no. NP_001003837.2). The putative polypeptide sequence shares 83% amino acid homology with human Src (GenBank accession no. NP_005408.1) with a conserved SH3 domain, SH2 domain and Tyrkc domain. The conservation scoring is performed by PRALINE (http://www.ibi.vu.nl/programs/pralinewww/). The scoring scheme works from 0 for the least conserved alignment position, up to 10 for the most conserved alignment position. The color assignments are blue represents unconserved to red represents conserved. (TIF) [file pone.0076951.s006.tif]

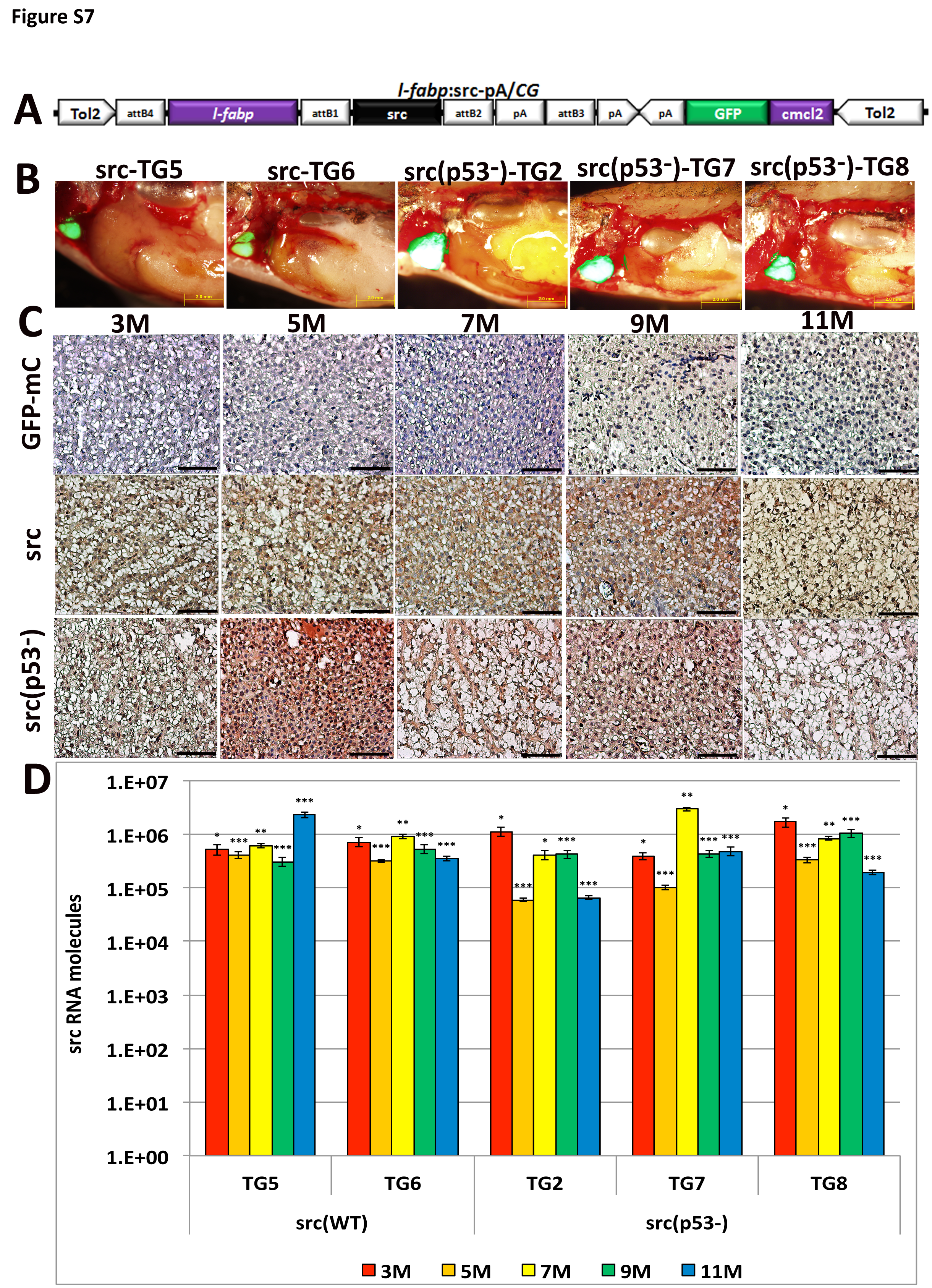

Supplement: Figure S7 — Generation and characterization of Tg (l-fabp:src-mC/CG) transgenic zebrafish. (A) Diagram of the l-fabp:src-mC/CG construct used in this study, containing Tol2 sequences, and the cmlc2:GFP expression cassette. (B) The src protein was expressed in the liver of wild-type and p53 mutant fish carrying the l-fabp:src-mC/CG transgene, as indicated by the cmlc2:GFP was expressed in the heart, as indicated by the green fluorescence. (C) (x 200) Immunohistochemical and (D) quantitative RT-PCR analysis of the expression of the src in hepatocytes from the 3, 5, 7, 9 and 11month old wild-type and p53 mutant fish overexpressing src and control l-fabp:GFP-mC transgenic fish. Scale bars: 50μm. (TIF) [file pone.0076951.s007.tif]
