# Supplementary material for: Liver-Specific Expressions of HBx and src in the p53 Mutant Trigger Hepatocarcinogenesis in Zebrafish
Source: PLoS One. 2013 Oct 9;8(10):e76951. doi: 10.1371/journal.pone.0076951 (PMC3793937; doi:10.1371/journal.pone.0076951)
Supplement: Table S1 — Summary of H&E stain revealed the pathology of liver tumor progression in GFP-mCherry, p53 mutant, HBx and src transgenic fish in wild-type and p53 mutant background, as well as the HBx+src double transgenic fish. (DOCX) [file pone.0076951.s008.docx]

**Table S1.**

| Type | Number of fish  stage | normal | Chronic inflammation | steatosis | bile duct dilation | hyperplasia | dysplasia | HCC | total case |
| --- | --- | --- | --- | --- | --- | --- | --- | --- | --- |
| GFP  (N=28) | 1.5M | 1 |  |  |  |  |  |  | 1 |
|  | 3M | 1 |  |  |  |  |  |  | 1 |
|  | 5M | 3 |  |  |  |  |  |  | 3 |
|  | 7M | 5 |  |  |  |  |  |  | 5 |
|  | 9M | 5 |  |  |  |  |  |  | 5 |
|  | 11M | 13 |  |  |  |  |  |  | 13 |
| p53^-^  (N=21) | 1.5M | 2 |  |  |  |  |  |  | 2 |
|  | 3M | 2 |  |  |  |  |  |  | 2 |
|  | 5M | 2 |  |  |  |  |  |  | 2 |
|  | 7M | 2 |  |  |  |  |  |  | 2 |
|  | 9M | 4 |  |  |  |  |  |  | 4 |
|  | 11M | 8 |  | 1 |  |  |  |  | 9 |
| HBx  (N=75) | 1.5M | 3 |  |  |  |  |  |  | 3 |
|  | 3M | 1 | 1 | 4 |  |  |  |  | 6 |
|  | 5M | 3 | 3 |  |  |  |  |  | 6 |
|  | 7M | 2 |  | 4 |  |  |  |  | 6 |
|  | 9M | 7 |  | 5 |  |  |  |  | 12 |
|  | 11M | 12 | 7 | 17 |  | 4 | 2 |  | 42 |
| HBx(p53^-^)  (N=53) | 1.5M |  |  | 2 |  |  |  |  | 2 |
|  | 3M |  | 2 |  |  | 1 |  |  | 3 |
|  | 5M |  |  | 1 |  |  | 1 |  | 2 |
|  | 7M | 1 | 1 | 3 |  | 1 |  |  | 6 |
|  | 9M |  | 1 | 1 |  |  | 2 |  | 4 |
|  | 11M | 2 | 8 | 4 |  |  | 6 | 16 | 36 |
| src  (N=47) | 3M |  |  | 4 |  |  |  |  | 4 |
|  | 5M |  |  | 4 |  |  |  |  | 4 |
|  | 7M | 1 |  | 3 |  |  |  |  | 4 |
|  | 9M |  |  | 5 |  |  | 1 |  | 6 |
|  | 11M | 6 | 2 | 11 | 2 | 1 | 2 | 5 | 29 |
| src(p53^-^)  (N=39) | 3M |  |  | 3 |  |  |  |  | 3 |
|  | 5M | 1 |  |  |  | 2 |  |  | 3 |
|  | 7M | 1 |  | 2 |  | 1 | 1 | 1 | 6 |
|  | 9M |  |  | 3 |  |  | 1 | 2 | 6 |
|  | 11M |  |  | 10 |  | 1 | 4 | 6 | 21 |
| HBx+src  (N=30) | 11M | 1 | 4 | 9 | 2 |  | 2 | 2 | 20 |
|  | 14M | 3 | 2 |  |  |  |  | 5 | 10 |
|  |  |  |  |  |  |  |  |  | 293 |
