# Supplementary material for: Liver-Specific Expressions of HBx and src in the p53 Mutant Trigger Hepatocarcinogenesis in Zebrafish
Source: PLoS One. 2013 Oct 9;8(10):e76951. doi: 10.1371/journal.pone.0076951 (PMC3793937; doi:10.1371/journal.pone.0076951)
Supplement: Table S2 — Summary of Sirius red Stain, PAS stain, TUNEL assay, Caspase3a and PCNA IHC results in GFP-mCherry, p53 mutant, HBx and src transgenic fish in wild-type and p53 mutant background. (DOCX) [file pone.0076951.s009.docx]

**Table S2.**

| Type | Age at sacrifice (months) | Gender | lines | Sirius red Stain (Score) | PAS stain (Score) | TUNEL assay (Score) | Caspase 3a (Score) | PCNA (Score) |
| --- | --- | --- | --- | --- | --- | --- | --- | --- |
| GFP-mC | 1.5M |  |  | 0 | 0 | 0 | 0 | 0 |
| p53^-^ | 1.5M |  |  | 0 | 0 | 0 | 1 | 0 |
| p53^-^ | 1.5M |  |  | 0 | 0 | 0 | 1 | 0 |
| HBx | 1.5M |  | G2 | 2 | 3 | 0 | 3 | 1 |
| HBx | 1.5M |  | G7 | 1 | 2 | 0 | 2 | 4 |
| HBx | 1.5M |  | G11 | 2 | 2 | 0 | 3 | 1 |
| HBx(p53^-^) | 1.5M |  | G2 | 0 | 3 | 2 | 3 | 1 |
| HBx(p53^-^) | 1.5M |  | G13 | 0 | 3 | 3 | 3 | 3 |
| GFP-mC | 3M |  |  | 0 | 2 | 0 | 0 | 0 |
| p53^-^ | 3M |  |  | 0 | 0 | 0 | 0 | 0 |
| p53^-^ | 3M |  |  | 0 | 0 | 0 | 1 | 0 |
| HBx | 3M |  | G2-1 | 2 | 4 | 1 | 2 | 2 |
| HBx | 3M |  | G2-2 | 1 | 4 | 1 | 2 | 2 |
| HBx | 3M |  | G7-1 | 1 | 4 | 2 | 1 | 2 |
| HBx | 3M |  | G7-2 | 1 | 1 | 0 | 3 | 2 |
| HBx | 3M |  | G11-1 | 1 | 3 | 2 | 2 | 2 |
| HBx | 3M |  | G11-2 | 0 | 2 | 2 | 2 | 2 |
| HBx(p53^-^) | 3M |  | G2-1 | 1 | 1 | 3 | 2 | 1 |
| HBx(p53^-^) | 3M |  | G2-2 | 2 | 3 | 3 | 4 | 1 |
| HBx(p53^-^) | 3M |  | G13 | 2 | 2 | 0 | 3 | 2 |
| src | 3M | F | G5-1 | 1 | 3 | 1 | 3 | 2 |
| src | 3M | F | G5-2 | 1 | 2 | 0 | 2 | 4 |
| src | 3M | F | G6-1 | 3 | 1 | 0 | 4 | 3 |
| src | 3M | F | G6-2 | 2 | 1 | 1 | 2 | 2 |
| src(p53^-^) | 3M |  | G2 | 1 | 2 | 1 | 2 | 3 |
| src(p53^-^) | 3M |  | G7 | 2 | 2 | 0 | 2 | 3 |
| src(p53^-^) | 3M |  | G8 | 1 | 3 | 0 | 2 | 3 |
| GFP-mC | 5M |  |  | 0 | 0 | 0 | 0 | 0 |
| GFP-mC | 5M |  |  | 0 | 0 | 0 | 0 | 0 |
| GFP-mC | 5M |  |  | 0 | 0 | 0 | 0 | 0 |
| p53^-^ | 5M |  |  | 0 | 1 | 0 | 1 | 0 |
| p53^-^ | 5M |  |  | 0 | 0 | 0 | 1 | 0 |
| HBx | 5M |  | G2-1 | 2 | 4 | 2 | 2 | 1 |
| HBx | 5M |  | G2-2 | 1 | 1 | 0 | 2 | 1 |
| HBx | 5M |  | G7-1 | 2 | 1 | 2 | 4 | 1 |
| HBx | 5M |  | G7-2 | 2 | 4 | 2 | 1 | 1 |
| HBx | 5M |  | G11-1 | 2 | 1 | 0 | 3 | 2 |
| HBx | 5M |  | G11-2 | 3 | 3 | 0 | 2 | 1 |
| HBx(p53^-^) | 5M |  | G2 | 3 | 1 | 1 | 2 | 1 |
| HBx(p53^-^) | 5M |  | G13 | 3 | 1 | 3 | 2 | 1 |
| src | 5M | F | G5-1 | 1 | 2 | 1 | 3 | 3 |
| src | 5M | F | G5-2 | 1 | 1 | 1 | 2 | 2 |
| src | 5M | M | G6-1 | 2 | 4 | 1 | 2 | 3 |
| src | 5M | M | G6-2 | 1 | 3 | 0 | 2 | 3 |
| src(p53^-^) | 5M |  | G2 | 0 | 2 | 0 | 2 | 2 |
| src(p53^-^) | 5M |  | G7 | 0 | 1 | 1 | 2 | 3 |
| src(p53^-^) | 5M |  | G8 | 1 | 3 | 1 | 2 | 2 |
| GFP-mC | 7M | F |  | 0 | 0 | 0 | 0 | 0 |
| GFP-mC | 7M | M |  | 0 | 1 | 0 | 0 | 0 |
| GFP-mC | 7M | M |  | 0 | 0 | 0 | 0 | 0 |
| GFP-mC | 7M | M |  | 0 | 1 | 0 | 0 | 0 |
| GFP-mC | 7M | M |  | 0 | 0 | 0 | 0 | 0 |
| p53^-^ | 7M |  |  | 0 | 2 | 0 | 0 | 0 |
| p53^-^ | 7M |  |  | 0 | 1 | 0 | 0 | 0 |
| HBx | 7M |  | G2-1 | 2 | 2 | 1 | 2 | 2 |
| HBx | 7M |  | G2-2 | 3 | 1 | 1 | 1 | 2 |
| HBx | 7M |  | G7-1 | 1 | 1 | 1 | 3 | 2 |
| HBx | 7M |  | G7-2 | 3 | 1 | 1 | 4 | 2 |
| HBx | 7M |  | G11-1 | 3 | 1 | 0 | 2 | 2 |
| HBx | 7M |  | G11-2 | 1 | 3 | 0 | 1 | 2 |
| HBx(p53^-^) | 7M | F | G2-1 | 3 | 1 | 1 | 4 | 2 |
| HBx(p53^-^) | 7M | F | G2-2 | 2 | 1 | 2 | 4 | 2 |
| HBx(p53^-^) | 7M | F | G2-3 | 0 | 1 | 1 | 3 | 2 |
| HBx(p53^-^) | 7M | F | G13-1 | 2 | 1 | 1 | 3 | 2 |
| HBx(p53^-^) | 7M | M | G13-2 | 2 | 1 | 1 | 2 | 2 |
| HBx(p53^-^) | 7M | M | G13-3 | 0 | 3 | 1 | 3 | 2 |
| src | 7M | F | G5-1 | 1 | 1 | 0 | 3 | 2 |
| src | 7M | F | G5-2 | 2 | 1 | 0 | 3 | 1 |
| src | 7M | M | G6-1 | 2 | 4 | 0 | 3 | 3 |
| src | 7M | M | G6-2 | 1 | 4 | 0 | 3 | 2 |
| src(p53^-^) | 7M | M | G2-1 | 2 | 2 | 2 | 3 | 2 |
| src(p53^-^) | 7M | F | G2-2 | 0 | 0 | 1 | 3 | 3 |
| src(p53^-^) | 7M | M | G7-1 | 3 | 4 | 3 | 0 | 2 |
| src(p53^-^) | 7M | M | G7-2 | 2 | 0 | 3 | 0 | 1 |
| src(p53^-^) | 7M | F | G8-1 | 0 | 0 | 0 | 1 | 1 |
| src(p53^-^) | 7M | F | G8-2 | 1 | 0 | 1 | 0 | 2 |
| GFP-mC | 9M | M |  | 0 | 1 | 0 | 0 | 0 |
| GFP-mC | 9M | M |  | 0 | 0 | 0 | 0 | 0 |
| GFP-mC | 9M | M |  | 0 | 0 | 0 | 0 | 0 |
| GFP-mC | 9M | F |  | 0 | 0 | 0 | 1 | 0 |
| GFP-mC | 9M | F |  | 0 | 0 | 0 | 0 | 0 |
| p53^-^ | 9M |  |  | 0 | 1 | 0 | 1 | 0 |
| p53^-^ | 9M |  |  | 0 | 2 | 0 | 0 | 0 |
| HBx | 9M | M | G2-1 | 3 | 4 | 0 | 2 | 3 |
| HBx | 9M | M | G2-2 | 3 | 4 | 1 | 3 | 1 |
| HBx | 9M | M | G2-3 | 3 | 3 | 0 | 2 | 1 |
| HBx | 9M | M | G2-4 | 2 | 3 | 1 | 1 | 1 |
| HBx | 9M | F | G7-1 | 3 | 1 | 2 | 3 | 2 |
| HBx | 9M | F | G7-2 | 3 | 2 | 0 | 3 | 2 |
| HBx | 9M | F | G7-3 | 3 | 1 | 0 | 2 | 1 |
| HBx | 9M | F | G7-4 | 1 | 2 | 0 | 3 | 2 |
| HBx | 9M | M | G11-1 | 2 | 2 | 0 | 1 | 2 |
| HBx | 9M | M | G11-2 | 2 | 4 | 1 | 2 | 2 |
| HBx | 9M | M | G11-3 | 2 | 3 | 2 | 1 | 2 |
| HBx | 9M | M | G11-4 | 1 | 4 | 1 | 1 | 1 |
| HBx(p53^-^) | 9M | F | G2-1 | 2 | 1 | 1 | 2 | 2 |
| HBx(p53^-^) | 9M | M | G2-2 | 1 | 3 | 2 | 3 | 2 |
| HBx(p53^-^) | 9M | F | G13-1 | 3 | 0 | 2 | 2 | 1 |
| HBx(p53^-^) | 9M | M | G13-2 | 0 | 2 | 2 | 2 | 1 |
| src | 9M | M | G5-1 | 2 | 0 | 0 | 0 | 1 |
| src | 9M | M | G5-2 | 1 | 0 | 0 | 0 | 0 |
| src | 9M | M | G5-3 | 1 | 0 | 1 | 0 | 1 |
| src | 9M | M | G6-1 | 3 | 4 | 3 | 0 | 0 |
| src | 9M | M | G6-2 | 0 | 3 | 0 | 0 | 1 |
| src | 9M | F | G6-3 | 0 | 2 | 0 | 3 | 0 |
| src(p53^-^) | 9M | M | G2-1 | 0 | 0 | 2 | 0 | 3 |
| src(p53^-^) | 9M | M | G2-2 | 0 | 2 | 0 | 0 | 0 |
| src(p53^-^) | 9M | F | G7-1 | 0 | 0 | 1 | 0 | 1 |
| src(p53^-^) | 9M | F | G7-2 | 0 | 3 | 0 | 0 | 0 |
| src(p53^-^) | 9M | M | G8-1 | 0 | 2 | 0 | 0 | 0 |
| src(p53^-^) | 9M | M | G8-2 | 0 | 2 | 2 | 0 | 0 |
| GFP-mC | 11M | F |  | 0 | 0 | 0 | 0 | 0 |
| GFP-mC | 11M | F |  | 0 | 0 | 0 | 0 | 0 |
| GFP-mC | 11M | M |  | 0 | 0 | 0 | 0 | 1 |
| GFP-mC | 11M | M |  | 0 | 1 | 0 | 1 | 0 |
| GFP-mC | 11M | M |  | 0 | 0 | 0 | 0 | 0 |
| p53^-^ | 11M |  |  | 0 | 0 | 0 | 0 | 0 |
| p53^-^ | 11M |  |  | 0 | 0 | 0 | 0 | 0 |
| HBx | 11M | F | G2-1 | 3 | 1 | 0 | 3 | 2 |
| HBx | 11M | M | G2-2 | 2 | 4 | 0 | 2 | 1 |
| HBx | 11M | M | G2-3 | 3 | 2 | 2 | 2 | 1 |
| HBx | 11M | M | G2-4 | 2 | 2 | 3 | 2 | 1 |
| HBx | 11M | F | G7-1 | 1 | 1 | 3 | 3 | 2 |
| HBx | 11M | F | G7-2 | 2 | 1 | 1 | 2 | 1 |
| HBx | 11M | F | G7-3 | 3 | 1 | 2 | 2 | 1 |
| HBx | 11M | M | G7-4 | 3 | 2 | 1 | 1 | 1 |
| HBx | 11M | M | G7-5 | 3 | 3 | 4 | 1 | 2 |
| HBx | 11M | M | G11-1 | 3 | 2 | 0 | 1 | 1 |
| HBx | 11M | M | G11-2 | 2 | 2 | 1 | 3 | 1 |
| HBx | 11M | M | G11-3 | 2 | 3 | 1 | 2 | 1 |
| HBx(p53^-^) | 11M | M | G2-1 | 0 | 3 | 0 | 2 | 3 |
| HBx(p53^-^) | 11M | M | G2-2 | 1 | 3 | 0 | 2 | 3 |
| HBx(p53^-^) | 11M | F | G2-3 | 0 | 0 | 0 | 2 | 4 |
| HBx(p53^-^) | 11M | M | G2-4 | 0 | 2 | 3 | 1 | 4 |
| HBx(p53^-^) | 11M | F | G2-5 | 0 | 0 | 0 | 1 | 3 |
| HBx(p53^-^) | 11M | F | G2-6 | 0 | 0 | 2 | 2 | 4 |
| HBx(p53^-^) | 11M | F | G2-7 | 0 | 1 | 1 | 2 | 2 |
| HBx(p53^-^) | 11M | F | G2-8 | 0 | 0 | 1 | 2 | 3 |
| HBx(p53^-^) | 11M | F | G2-9 | 0 | 1 | 0 | 2 | 1 |
| HBx(p53^-^) | 11M | M | G13-1 | 1 | 2 | 0 | 2 | 2 |
| HBx(p53^-^) | 11M | M | G13-2 | 0 | 2 | 1 | 2 | 1 |
| HBx(p53^-^) | 11M | F | G13-3 | 0 | 0 | 1 | 2 | 3 |
| HBx(p53^-^) | 11M | F | G13-4 | 0 | 1 | 1 | 4 | 3 |
| HBx(p53^-^) | 11M | F | G13-5 | 0 | 1 | 0 | 3 | 3 |
| HBx(p53^-^) | 11M | F | G13-6 | 2 | 1 | 0 | 3 | 1 |
| HBx(p53^-^) | 11M | F | G13-7 | 0 | 1 | 3 | 3 | 4 |
| HBx(p53^-^) | 11M | F | G13-8 | 2 | 1 | 2 | 2 | 4 |
| src | 11M | F | G5-1 | 0 | 0 | 0 | 3 | 3 |
| src | 11M | F | G5-2 | 0 | 1 | 1 | 3 | 3 |
| src | 11M | F | G5-3 | 0 | 0 | 0 | 3 | 2 |
| src | 11M | M | G5-4 | 0 | 2 | 0 | 2 | 1 |
| src | 11M | M | G6-1 | 0 | 2 | 0 | 2 | 2 |
| src | 11M | M | G6-2 | 0 | 1 | 0 | 1 | 1 |
| src | 11M | M | G6-3 | 0 | 2 | 0 | 1 | 1 |
| src | 11M | M | G6-4 | 0 | 2 | 0 | 1 | 3 |
| src | 11M | M | G6-5 | 0 | 2 | 0 | 1 | 2 |
| src(p53^-^) | 11M | M | G2-1 | 1 | 0 | 0 | 0 | 3 |
| src(p53^-^) | 11M | F | G2-2 | 1 | 0 | 0 | 0 | 0 |
| src(p53^-^) | 11M | F | G7-1 | 0 | 0 | 0 | 0 | 1 |
| src(p53^-^) | 11M | M | G7-2 | 1 | 2 | 0 | 0 | 0 |
| src(p53^-^) | 11M | F | G8 | 1 | 2 | 1 | 1 | 1 |
