# Supplementary material for: Liver-Specific Expressions of HBx and src in the p53 Mutant Trigger Hepatocarcinogenesis in Zebrafish
Source: PLoS One. 2013 Oct 9;8(10):e76951. doi: 10.1371/journal.pone.0076951 (PMC3793937; doi:10.1371/journal.pone.0076951)
Supplement: Table S3 — Primer sequence. (DOCX) [file pone.0076951.s010.docx]

**Table S3.**

| Gene Name | Primer name | Start | Sequence (5’ to 3’) | Accession number | Size (bp) |
| --- | --- | --- | --- | --- | --- |
| **Gateway recombinationtransgenesis** | | | | | |
| *l-fabp* | attB4-*l-fabp*-F |  | GGGGACAACTTTGTATAGAAAAGTTGTCGATCTGCTGCAGTTCGAACT | NM_001044712.1 |  |
|  | attB4-*l-fabp*-F1 |  | GGGGACAACTTTGTATAGAAAAGTTGTCGATCTGCTGCAGTTCGAACT |  |  |
|  | attB1r-*l-fabp*-R |  | GGGGACTGCTTTTTTGTACAAACTTGGCTTTCTGGAGAAGCTCAACA |  |  |
| *HBx* | attB1-*HBx*-F | 1 | GGGGACAAGTTTGTACAAAAAAGCAGGCTATGGCTGCTAGGCTGTGCTG | U95551.1 | 463 |
|  | attB2-*HBx*-R | 463 | GGGGACCACTTTGTACAAGAAAGCTGGGTTAGGCAGAGGTGAAAAAGTTGC |  |  |
| *src* | attB1-*src*-F | 1 | GGGGACAAGTTTGTACAAAAAAGCAGGCTATGGGTGGAGCCAAGAGTAA | NM_001003837.2 | 1605 |
|  | attB2-*src*-R | 1605 | GGGGACCACTTTGTACAAGAAAGCTGGGTCTAGAGGTTTTCTCCGGGTTGG |  |  |
| **lipogenic factors** | | | | | |
| *pparg* | Q-*pparg*-F | 513 | GGTTTCATTACGGCGTTCAC | NM_131467.1 | 250 |
|  | Q-*pparg*-R | 762 | TGGTTCACGTCACTGGAGAA |  |  |
| *srebf1* | Q-*srebf1*-F | 2163 | CATCCACATGGCTCTGAGTG | NM_001105129.1 | 250 |
|  | Q-*srebf1*-R | 2412 | CTCATCCACAAAGAAGCGGT |  |  |
| *chrebp* | Q-*chrebp*-F-2 | 818 | GGAGATGGACTCGCTCTTTG | XM_001338467 | 200 |
|  | Q-*chrebp*-R-2 | 513 | GCAGAGGCTCAAAAGTGTCC |  |  |
| **lipogenic enzyme** | | | | | |
| *fasn* | Q-*fasn*-F | 7183 | ATCTGTTCCTGTTCGATGGC | XM_682295 | 250 |
|  | Q-*fasn*-R | 7432 | AGCATATCTCGGCTGACGTT |  |  |
| *pap* | Q-*pap*-F | 976 | CAGTTCTTCCTGATTGCTGC | XM_692415 | 250 |
|  | Q-*pap*-R | 1225 | TCCTCAAAGCTTAGTTCGGG |  |  |
| *dgat2* | Q-*dgat2*-F | 522 | TGGGGCTTTTTGTAACTTCG | NM_001030196 | 250 |
|  | Q-*dgat2*-R | 771 | TCTTCCTGGTGCACAGTCC |  |  |
| **pparg target** | | | | | |
| *ucp2* | Q-*ucp2*-F | 613 | GAGCTTTGCTTCTGTACGCA | NM_131176 | 250 |
|  | Q-*ucp2*-R | 862 | ACGAAACCCCTCTTCCTTTG |  |  |
| *cfdl* | Q-*cfdl*-F-2 | 245 | GGTTGTTTTGGGTGCTCACT | NM_001020532.1 | 199 |
|  | Q-*cfdl*-R-2 | 443 | TTTAGGGTCAGCCGTCTCAT |  |  |
| *adipoql2* | Q-*adipoql2*-F | 337 | AGGATTTCCAGGCAAAAGAG | XM_685982.5 | 250 |
|  | Q-*adipoql2*-R | 586 | GCCATTGATGGTGAGGTGAT |  |  |
| **lipid beta-oxidation genes** | | | | | |
| *pparab* | Q-*pparab*-F | 221 | CGTCGTCAGGTGTTTACGGT | NM_001102567.1 | 250 |
|  | Q-*pparab*-R | 470 | AGGCACTTCTGGAATCGACA |  |  |
| *cpt1* | Q-*cpt1*-F | 1470 | ACTCTCGATGGACCCTGTGA | NM_001044854 | 250 |
|  | Q-*cpt1*-R | 1719 | CTGGATGAAGGCATCTGGAC |  |  |
| *l-pbe* | Q*-l-pbe*-F | 1412 | GGAAGGTGAGTGTGGCAGTT | NM_207068 | 250 |
|  | Q*-l-pbe*-R | 1661 | GGAGGATCTTTGGGGTCAAC |  |  |
| *cyp4a10* | Q-*cyp4a10*-F | 324 | CATTCTCACAACCACAGAAC | XM_001331436 | 206 |
|  | Q-*cyp4a10*-R | 529 | GAACTTCCCATTTATCAAGC |  |  |
| *acox3* | Q-*acox3*-F | 481 | AAGGACATCGAGCGAATGAT | NM_213147 | 250 |
|  | Q-*acox3*-R | 730 | CTATGAAAGAGTGGAGGCCG |  |  |
| **cell cycle/division related genes** | | | | | |
| *ccna1* | Q-*ccna1*-F | 151 | TTGTGCTTGGTGTTTTGACC | NM_212818.1 | 197 |
|  | Q-*ccna1*-R | 347 | TAGCAGTTCTGAAGGCAGCA |  |  |
| *ccnb1* | Q-*ccnb1*-F | 603 | GCGTGCCATTCTTATCGACT | NM_131513.1 | 199 |
|  | Q-*ccnb1*-R | 801 | TGCAATCTCTGGTGGGTACA |  |  |
| *ccne1* | Q-*ccne1*-F | 371 | TCCCGACACAGGTTACACAA | NM_130995.1 | 201 |
|  | Q-*ccne1*-R | 571 | TTGTCTTTTCCGAGCAGGTT |  |  |
| *ccng1* | Q-*ccng1*-F | 603 | GCTCAACTGGAAGGTCAAGG | NM_199481.1 | 199 |
|  | Q-*ccng1*-R | 801 | CAGGGCCAGAAGAGACAAAG |  |  |
| *cdk1* | Q-*cdk1*-F | 779 | CTCTGGGGACCCCTAACAAT | NM_212564.2 | 200 |
|  | Q-*cdk1*-R | 978 | CGGATGTGTCATTGCTTGTC |  |  |
| *cdk2* | Q-*cdk2*-F | 794 | CAGCTCTTCCGGATATTTCG | NM_213406.1 | 199 |
|  | Q-*cdk2*-R | 992 | CCGAGATCCTCTTGTTTGGA |  |  |
| **fibrosis marker genes** | | | | | |
| *col1a1a* | Q-*col1a1a*-F | 2811 | TATTGGTGGTCAGCGTGGTA | NM_199214.1 | 199 |
|  | Q-*col1a1a*-R | 3009 | TCCTGGAGTACCCTCACGAC |  |  |
| *ctgfa* | Q-*ctgfa*-F | 481 | TGTGTGTTTGGTGGAATGGT | NM_001015041.2 | 198 |
|  | Q-*ctgfa*-R | 678 | GGAGTCACACACCCACTCCT |  |  |
| *hpse* | Q-*hpse*-F | 713 | GCTCTGGTTTGGAGCTCATC | NM_001045005.1 | 203 |
|  | Q-*hpse*-R | 915 | GAAATCCCGACCAAGTTGAA |  |  |
| **Metastasis markers** | | | | | |
| *mmp2* | Q-*mmp2*-F | 191 | TCTTGCTTCCCTGCAAACTT | NM_198067.1 | 209 |
|  | Q-*mmp2*-R | 399 | GGTCAATCTCCCCTGTCTCA |  |  |
| *timp2a* | Q-*timp2a*-F | 327 | CGTTCTGCAATGCTGATGTT | NM_182874.1 | 202 |
|  | Q-*timp2a*-R | 528 | TCCAAATTGGTCACTCCACA |  |  |
| **tumor markers** | | | | | |
| *p53* | Q-*p53*-F | 597 | TTGTCCCATATGAAGCACCA | NM_131327.1 | 200 |
|  | Q-*p53*-R | 796 | TTTCCTGTCTCTGCCTGGAC |  |  |
| *ccnd1* | Q-*ccnd1*-F | 769 | TTCCTTGCCAAACTGCCTAT | NM_131025.4 | 201 |
|  | Q-*ccnd1*-R | 969 | GGTGAGGTTCTGGGATGAGA |  |  |
| *mycb* | Q*-mycb-F* | 688 | GGTGTTTCCCTTTCCACTGA | NM_200172.1 | 197 |
|  | Q*-mycb-R* | 884 | TTCTCTTTTCCACCGTGACC |  |  |
| **HBx, src and internal control** | | | | | |
| *HBx* | Q-*HBx*-F | 264 | GTTGCCCAAGGTCTTACATA | U95551.1 | 157 |
|  | Q-*HBx*-R | 420 | TTTATGCCTACAGCCTCCTA |  |  |
| *src* | Q-*src*-F | 148 | ACACAGCCCAACATCATCAA | NM_001003837.2 | 248 |
|  | Q-*src*-R | 395 | TATCCGCTCTCTCCTGTCGT |  |  |
| *18s* | Q-*18s*-F2 | 551 | GAGAAACGGCTACCACATCC | XM_001922869.1 | 169 |
|  | Q-*18s*-R2 | 719 | ACCAGACTTGCCCTCCAA |  |  |
| *actin* | Q-*actin* -F | 893 | CTCCATCATGAAGTGCGACGT | NM_131031.1 | 180 |
|  | Q-*actin* -R | 1072 | CAGACGGAGTATTTGCGCTCA |  |  |
