# Supplementary material for: Liver-Specific Expressions of HBx and src in the p53 Mutant Trigger Hepatocarcinogenesis in Zebrafish
Source: PLoS One. 2013 Oct 9;8(10):e76951. doi: 10.1371/journal.pone.0076951 (PMC3793937; doi:10.1371/journal.pone.0076951)
Supplement: Experimental Procedures S1 — The detail information for material and methods are all described in this supporting information file (DOC) [file pone.0076951.s011.doc]

**Experimental Procedures S1**

**Zebrafish maintenance**

Zebrafish were maintained at the Zebrafish Core Facility at NTHU-NHRI (ZeTH) according to established protocols . The zebrafish embryos, larvae, and adult fish were maintained at 28°C under continuous flow in the zebrafish core facility with automatic control of a 14 hour light and 10 hour dark cycle. All experiments involving zebrafish were approved by the Institutional Animal Care and Use Committee (IACUC) of the NHRI (NHRI-IACUC-101005-A).

**Generation of transgenic zebrafish using the Tol2 transposon system**

The zebrafish *fabp* promoter was amplified using the attB4-*l-fabp*-F and attB1r-*l-fabp*-R primers from the p*l-fabp*-tTA plasmid template . The HBx gene was amplified using the primer pair of attB1-HBx-F and attB2-HBx-R and the Alb-2HS4/HBx plasmid as template . The zebrafish *src* gene was amplified using the primer pair of attB1-*src*-Fand attB2-*src*-R primers and the 24 hours cDNA as template. The primer sequences are listed in Supplementary Table 1. The construction of the destination plasmids was carried out according to the manufacturer’s recommendations (MultiSite Gateway® Three-Fragment Vector Construction Kit, Invitrogen, Grand Island, NY, USA). All plasmid constructs were confirmed by sequencing. The transgenic founders were created using the Tol2 transposon system as described previously .

**Liver tissue collection and paraffin sectioning**

HBx transgenic fish were euthanized at 1.5, 3, 5, 7, 9, and 11 months of age, and src transgenic fish were euthanized at all stages listed above except 1.5 months. The tissues were frozen in liquid nitrogen immediately after dissection and stored at -70°C for later RNA extraction. For histochemistry, liver tissues were fixed in a 10% formalin solution (Sigma-Aldrich, St. Louis, MO, USA). The fixed tissue was embedded in paraffin, sectioned in five-micrometer thicknesses, and mounted on poly-L-lysine coated slides. Following deparaffinization, sections were stained with hematoxylin and eosin. All samples were assessed by three trained pathologists: Dr. Yueh-Min Lin, Changhua Christian Hospital, Changhua, Taiwan; Dr. Pen-Heng Chang who is a fish pathologist from Department of Veterinary Medicine, National Taiwan University, and. Dr. Jim-Ray Chen who is fish pathologist expertise at liver cancer from Department of Pathology, Chang Gung Memorial Hospital.

**Immunohistochemistry analyses**

For immunohistochemistry, the sections were incubated at 4^o^C overnight with 1:100 dilutions of primary antibodies which included mouse anti-HBx (**Abcam,** Cambridge, MA, USA), rabbit anti-active caspase 3 (**BD Biosciences,** San Jose, CA, USA), mouse anti-PCNA (**Santa Cruz Biotechnology, Inc.,** Santa Cruz, CA, USA), rabbit anti-SRC (GeneTex Inc., Hsinchu City, Taiwan)**,** rabbit anti-pERK1/2 (Cell Signaling Technology, Danvers, MA, USA**),** rabbit anti-pAKT (Cell Signaling Technology, Danvers, MA, USA**),** rabbit anti-Myc (AnaSpec Inc., San Jose, CA, USA**),** rabbit anti-VEGF (AnaSpec Inc., San Jose, CA, USA**),** rabbit anti-JNK1 (AnaSpec Inc., San Jose, CA, USA), mouse anti-phospho-Src (Tyr416) (Millipore, Temecula, CA), rabbit anti-phospho-Src (Tyr418) (Millipore, Temecula, CA), and rabbit anti-phospho-Src (Tyr527) (Cell Signaling, Danvers, MA, USA). After washing with PBS, the sections were incubated with a 1:100 dilution of the secondary antibodygoat anti-rabbit IgG (Santa Cruz Biotechnology, Inc.) or goat anti-mouse IgG (Santa Cruz Biotechnology, Inc.) at room temperature, followed by development using the Liquid DAB Substrate Kit (Invitrogen, Carlsbad, CA, USA). The multiple cancer tissue array (catalogue#:MC801) and multiple normal tissue array (catalogue#:BN1002a) were purchased from US Biomax, Inc. (Rockville, Maryland, USA). Diagnosis was carried out by single-blind evaluation of all samples by three trained pathologists.

**Sirius red staining, Oil-red staining, Periodic Acid-Schiff Stain, TUNEL assay, Immunostaining of anti-caspase 3 and PCNA**

Sirius red staining was performed using the Picrosirius Red Stain Kit (Polysciences, Inc., Warrington, PA, USA) to detect collagen fibers. Glycogen accumulation was detected using the Periodic Acid-Schiff Stain Kit (Polysciences, Inc.). The liver tissues were also subjected to a terminal deoxynucleotidyl transferase dUTP nick end labeling (TUNEL) assayusing the *In Situ* Cell Death Detection Kit (Fluorescein, Roche). Cell apoptosis was examined by immunostaining with an anti-caspase 3 (active) antibody. Cell proliferation was checked using an anti-proliferating cell nuclear antigen (PCNA) antibody for immunostaining. The tissue sections were dehydrated, cleared, mounted and examined using light microscopy. The Sirius Red staining and TUNEL assay results were scored by staining intensity, ranging from negative (0) to minimal (1), moderate (2), and maximal (3) (Supplementary Figure 3A and B). The PAS, caspase 3a and PCNA staining were evaluated by the percentage of expression, with scores 0 to 4 representing 0-15%, 16-25%, 26-50%, 51-75% and 76-100% expression (Supplementary Figure 3 C-E). Lipid was detected by Oilred staining. Frozen liver sections were washed three times with PBS, followed by fixation with 10% formalin in phosphate buffer for 1 h at room temperature. After fixation, the samples were again washed with PBS and then stained with a filtered Oil red O (Sigma-Aldrich) solution (0.3 g Oil red O in 100 ml isopropanol) for 2 hrs at room temperature. The samples were then washed twice with 60% isopropanol for 15 min and nuclei stained with hematoxylin (Fig. S2).

**RNA isolation and quantitative RT-PCR**

The liver tissues were frozen in liquid nitrogen immediately after dissection and stored at -70°C for subsequent RNA extraction. The total RNA from the liver tissue was isolated using the RNA Spin Mini RNA Isolation Kit (GE Healthcare) and reverse transcribed using the SuperScript II cDNA Synthesis Kit (Invitrogen). Quantitative RT-PCR (Q-PCR) was performed using the SYBR Green Q-PCR Master Mix Kit (Applied Biosystems, Carlsbad, CA) using an ABI PRISM 7900 System. Gene expression in each sample was normalized to the expression level of β-actin as an internal control. Each Q-PCR was performed in triplicate, and the medians were calculated. At least three independent samples were used for Q-PCR, and the data are expressed as the median ± standard error. To quantify the absolute amounts of RNA, serial dilutions of the cDNA fragments were used in Q-PCR to create a standard curve as described previously . Differences among variables were assessed by a two-tailed Student’s *t* test. A P<0.05 was considered statistically significant and is shown as: *: 0.01<P≤0.05; **: 0.001<P≤0.01; and ***: P≤0.001. The sequences of the primers used for q-RT-PCR are provided in Supplementary Table 1.

**Western blot analysis**

Livers were harvested and lysed in RIPA buffer with the addition of protease inhibitor cocktail (Roche Molecular Biochemicals, Mannheim, Germany). Total protein concentrations were determined by Bradford protein assay (Bio-Rad, Hercules, CA). Ten micrograms of total protein of each sample was loaded per well for SDS-PAGE and transferred to PVDF membrane. Antibodies against signaling molecules including Erk (#9102), phospho-Erk (#4370), Akt (#9272), and phospho-Akt (#9271) were purchased from Cell Signaling Technology (1:1000 dilution for Erk and phospho-Erk, 1:500 for Akt and phospho-Akt; Danvers, MA). Anti-β-actin antibody (A5316, 1:5000 dilution; Sigma-Aldrich, St. Louis, MO) was used for the internal control. Mouse or rabbit secondary antibody conjugated with HRP (1:2000 dilution; Santa Cruz Biotechnology, Santa Cruz, CA) was applied. The chemiluminescentsignals were visualized by Western Lightning Plus-ECL reagent (Perkin Elmer, Waltham, MA) and captured by the BioSpectrumAC Imaging System (UVP, Upland, CA). Band intensity was quantified by UVP Visionworks LS software.

**Statistics**

The unpaired Student's t test, Kaplan-Meier analysis, ANOVA test and two-tailed Fisher exact test were used in the data analysis as described earlier . Cumulative frequency of the pathology changes in transgenic zebrafish was calculated by Kaplan-Meier analysis . Statistical analysis of Q-PCR, tissue microarray and cumulative frequency results were performed using the unpaired Student's t test, ANOVA test or with two-tailed Fisher exact test. A p-value of less than 0.05 was considered to be statistically significant.
